# Modulating the G‐Quadruplex and Duplex DNA Binding by Controlling the Charge of Fluorescent Molecules

**DOI:** 10.1002/chem.202203094

**Published:** 2022-12-08

**Authors:** Ariadna Gil‐Martínez, Sònia López‐Molina, Cristina Galiana‐Roselló, Andrea Lázaro‐Gómez, Friederike Schlüter, Fabio Rizzo, Jorge González‐García

**Affiliations:** ^1^ Institute of Molecular Science (ICMol) Department of Inorganic Chemistry University of Valencia Catedrático José Beltrán 2 46980 Paterna Spain; ^2^ Center for Soft Nanoscience (SoN) Westfälische Wilhelms-Universität Münster Busso-Peus-Str. 10 48149 Münster Germany; ^3^ Istituto di Scienze e Tecnologie Chimiche (SCITEC) Consiglio Nazionale delle Ricerche (CNR) via G. Fantoli 16/15 20138 Milano Italy

**Keywords:** duplex DNA, fluorescence, G-quadruplex, spirobifluorene, triphenylamine

## Abstract

Two fluorescent and non‐toxic spirobifluorene molecules bearing either positive (Spiro‐NMe3) or negative (Spiro‐SO3) charged moieties attached to the same aromatic structure have been investigated as binders for DNA. The novel Spiro‐NMe3 containing four alkylammonium substituents interacts with G‐quadruplex (G4) DNA structures and shows preference for G4s over duplex by means of FRET melting and fluorescence experiments. The interaction is governed by the charged substituents of the ligands as deduced from the lower binding of the sulfonate analogue (Spiro‐SO3). On the contrary, Spiro‐SO3 exhibits higher binding affinity to duplex DNA structure than to G4. Both molecules show a moderate quenching of the fluorescence upon DNA binding. The confocal microscopy evaluation shows the internalization of both molecules in HeLa cells and their lysosomal accumulation.

## Graphical abstract

**Two fluorescent** and non‐toxic spirobifluorene molecules being either positive (Spiro‐NMe3) or negative (Spiro‐SO3) charged have been investigated as DNA binders. Both molecules show a moderate binding to G‐quadruplex and duplex DNA structures whereas the interaction is governed by the overall charge of the ligands.

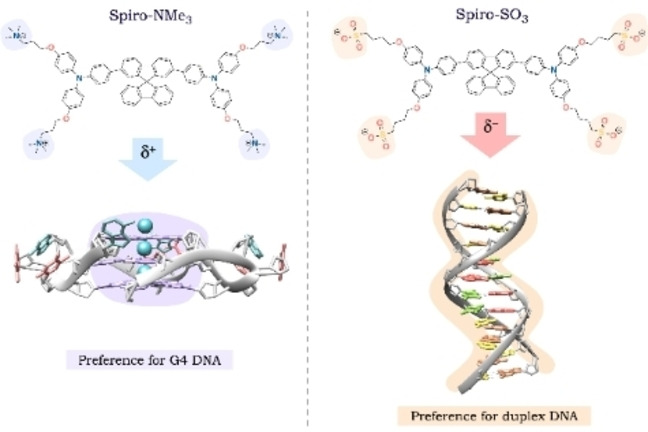

## Introduction

**Figure 1:**
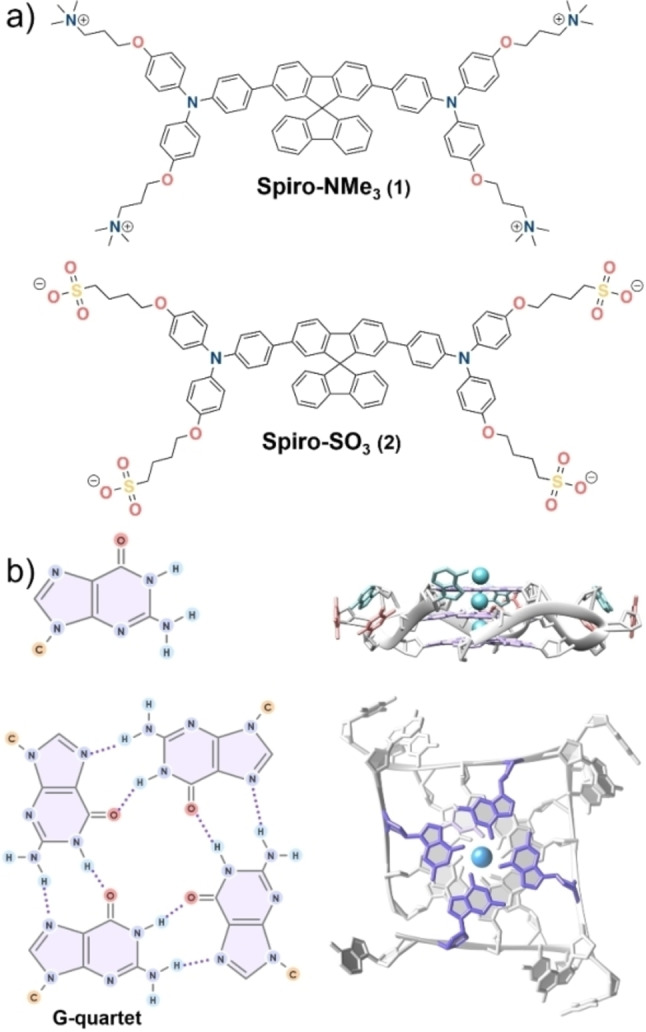
(a) Ligands studied in this work. (b) Structure of guanine, schematic illustration of a G‐tetrad and schematic illustration of typical intramolecular G4 structure (PDB code: 1KF1). PDB; Protein Data Bank.

Organisms store the genetic information in polymeric molecules of nucleotides either as DNA or RNA. In particular, DNA has been associated to the structure determination by Watson and Crick of the double‐stranded helix with the essential crystallographic assistance of Wilkins, Franklin and many others in the 1950s. Nevertheless, DNA can adopt transient and permanent alternative conformations such as triplexes, Holliday junctions, *i*‐motifs and G‐quadruplexes (G4s) assisted by the capability of nucleobases to form different hydrogen bond patterns from the Watson‐Crick base pairing.^[1]^ G4s are tetra‐stranded structures formed from both DNA and RNA guanine‐rich sequences. These structures are built from two or more guanine‐quartet units stacked with each other. G‐quartets or G‐tetrads are planar rearrangements of four guanine bases hold together by a hydrogen bonding network between the Hoogsteen and Watson‐Crick faces of the guanines (Figure 1).^[2]^ G‐quadruplex frameworks retain cationic ions in a central core channel, being sodium and potassium ions the most relevant from the biological point of view. Striking, next generation sequencing and bioinformatic analysis identified putative G‐quadruplex forming sequences (*ca*. >700 000) in human telomeres, oncogene‐promoter regions, replication initiation sites and untranslated regions in the human genome.^[3]^ The accumulating evidences highlight the essential role of G‐quadruplexes in gene expression, telomere maintenance and chromosome stability.^[4]^ In consequence, G4s have been proposed as potential targets for the therapeutic intervention in cancer, aging and neurodegenerative diseases,^[5]^ and a large number of small molecules have been described to exert a therapeutic benefit.^[6]^ In this line, some G4 binders have progressed to clinical trials for the treatment of carcinoid, neuroendocrine and BRCA1/2‐defficient tumors.^[7]^ In parallel to the therapeutical intervention, many biological tools have been developed to investigate G4s *in vitro* and *in vivo*.^[8]^ Among them, optical probes are of outmost importance to track the G4 formation and to understand their regulatory roles in the biological processes. The chimeric fluorescent probe shall own several features, (i) high‐to‐moderate affinity for G4s, (ii) selectivity for G4s over other DNA/RNA structures and proteins present in the cells, (iii) large variation of the fluorescence upon G4 binding, (iv) negligible change in fluorescence upon interaction with other biomolecules except for G4s, (v) permeable to cells and (vi) to not be toxic.^[9]^ Therefore, the chemical biologist community is seeking for optical probes to interrogate the G‐quadruplex formation in the biological processes of living systems. Some examples of optical probes are collected in recent reviews and include both organic molecules and metal complexes.^[10]^ In the pursuit to find new scaffolds to detect G4s, triphenylamine derivatives have been developed to detect G4s based on an aggregation process between the molecules and the tetrameric G4 structures, generating an aggregation induced emission band.^[11]^ Moreover, G4s detection has been achieved by using probes based on the change in the fluorescence lifetime upon binding to different DNA structures.^[12]^

However, many fluorescent probes exhibiting selectivity towards either G4 or duplex DNA structures require different molecular design, which results in a remarkable synthetic effort.^[13]^ Indeed, the probes reported in literature are designed to show exclusive binding affinity, while examples of emitting aromatic systems that can be employed to discern between G‐quadruplex and duplex structures by means of an easy chemical modification are scarcely reported.

In this work, we propose an easy synthetic approach to obtain water soluble fluorophores able to distinguish between G4 and duplex DNA by modulating the charge introduced as lateral substituents in an aromatic core. In particular, we have focused on the tetragonal‐shaped scaffold, namely the spirobifluorene, as a potential unit to bind G4s, which is unexplored as nucleic acids binder. Spirobifluorene has shown potential for optoelectronic devices because of its unique conjugated cross‐shape structure that hampers the aggregation, thus reducing the quenching of the emission from a non‐radiative decay pathway.^[14]^ The chemical versatility of the spirobifluorene core allows modifying the photophysical properties, resulting in many different applications.^[15]^ Recently, we reported on the synthesis of the first highly water soluble spirobifluorene derivative Spiro‐SO3 (**2**, see Figure 1)^[16]^ showing very low cytotoxicity and cellular uptake.

Herein, we designed and synthesized a new spirobifluorene derivative (Spiro‐NMe3 (**1**), Figure 1) containing four trimethylamine pendant substituents and unambiguously characterized the structure by NMR and mass spectrometry. The photophysical properties of the Spiro‐NMe3 (**1**) and the sulfonate analogue, Spiro‐SO3 (**2**), were investigated. Moreover, we assessed the interaction of both ligands towards a panel of G4 and duplex structures by using FRET melting and fluorescence studies. Furthermore, the selectivity of Spiro‐NMe3 (**1**) towards G4 s over duplex was investigated via FRET melting competition assays and molecular docking. To assess the potential sensing application of the ligands in cells, we determined the viability in HeLa cells and monitored the luminescence of the ligands in the cells in tandem with co‐localization probes to get insights in which cellular compartment the ligands are accumulated.

## Results and Discussion

### Design, synthesis and photophysical properties of spirobifluorene molecules

We initially selected the spirobifluorene core in our G4‐ligand design because it adopts a rigid tetragonal‐shaped aromatic conformation, which presumably impacts in the ability to stack on the G‐tetrads and/or fit into the grooves. We conjugated this core to two triphenylamine units at the 2,7 positions of the spirobifluorene ring in order to increase the π‐π stacking interactions as well as allow these units to aggregate, which can cause a change in the fluorescence emission. Finally, we envisaged that four pendant arms in the structural design with ionizable groups will increase the aqueous solubility and can form electrostatic interactions with the DNA backbone or bases. We selected either the sulfonate and the tetraalkylammonium groups as anionic and cationic moieties to investigate the net charge of the molecules in the interaction, −4 and +4 respectively.

**Scheme 1:**
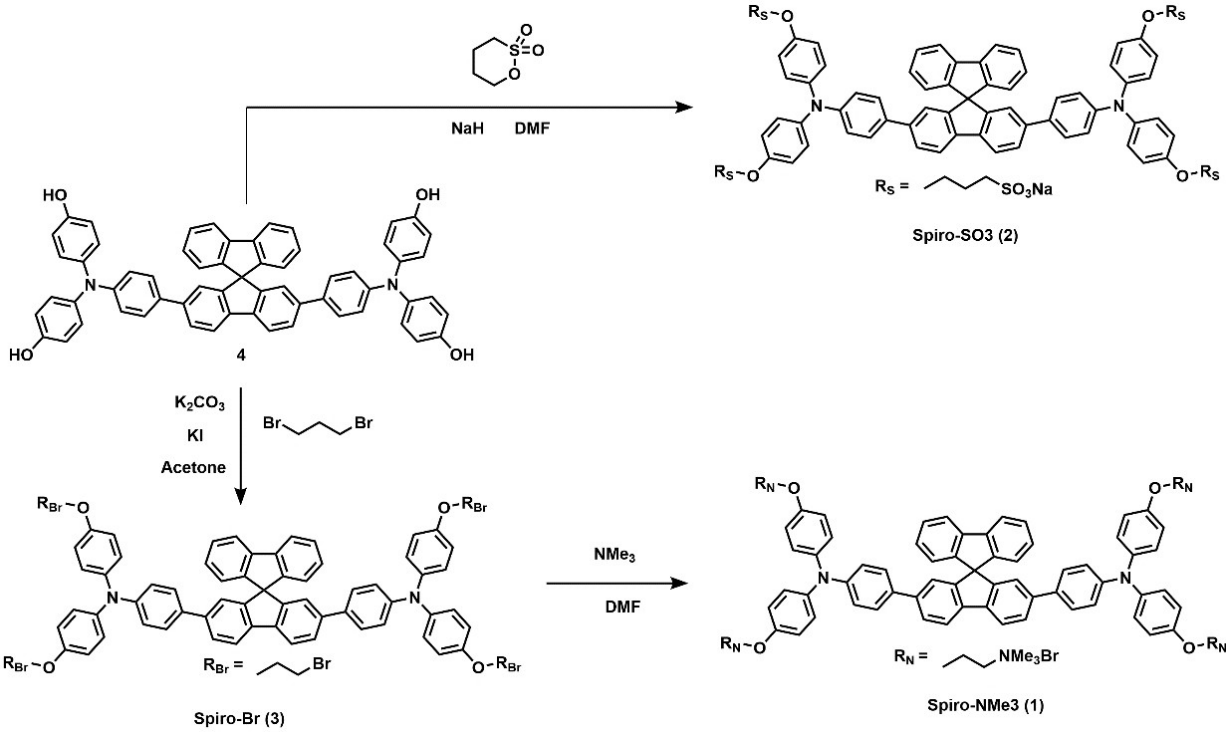
Synthetic route of spirobifluorene ligands studied in this work.

The preparation of the organic dyes is carried out by following the same approach, that is, firstly by preparing the aromatic core^[17]^ followed by the introduction of the lateral alkyl chains (see Scheme 1). By starting from the aromatic intermediate bearing four hydroxy groups (**4** in Scheme 1), it was possible to introduce directly the chain ending with sulfonate by reaction with 1,4‐butansultone in presence of sodium hydride to afford the Spiro‐SO3 (**2**).^[16]^ Spiro‐NMe3 (**1**) was obtained through two synthetic steps, that is, firstly introducing the alkyl chain by reaction with 1,3‐dibromopropane in presence of K_2_CO_3_ as base and a catalytic amount of KI to give the intermediate **3** (Scheme 1 and Figures S1‐S3 in Supporting Information), followed by the reaction with an excess of trimethylamine to give the tetraalkylammonium bromide salt (see Figures S4–S8 for NMR and mass spectra). Our synthetic strategy allows modulating easily the electrostatic interactions between the dye and the DNA by keeping the same aromatic core.

Both molecules contain the same spirobifluorene unit as aromatic scaffold, which results in similar absorption spectra with the maxima of the π‐π* band around 380 nm (see Table S1 in Supporting Information) and a second absorption band centered at 290 nm attributed to n‐ π* transitions derived from the triphenylamine units.^[18]^ In contrast, the photoluminescence in water (PL) is slightly different among the molecules, showing a band centered at 480 nm for SpiroSO3 (**2**) while the maxima of the emission band for the cationic dye is red‐shifted up to 505 nm (see Figures S9 and S10). The wavelength variation could likely arise from the different ligand solvation as consequence of the different molecular net charge and the different length of the alkyl chains.^[16]^

### Fluorescence Resonance Energy Transfer (FRET) melting assays

**Figure 2:**
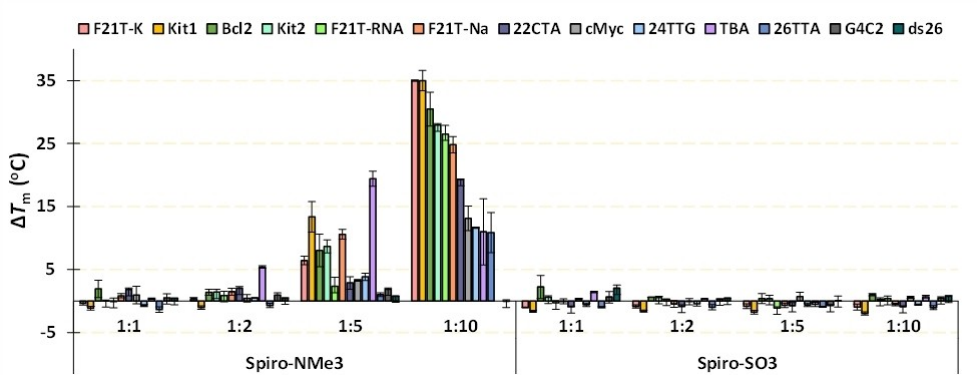
Representation of FRET melting values (Δ*T*_m_) for the interaction between spirobifluorenes and various G4s and a duplex. The concentration of DNA was 0.2 μm and the [DNA]:[Ligand] ratios were presented in the bottom part (1 : 1, 1 : 2, 1 : 5 and 1 : 10). Errors denote the standard deviations of at least three independent experiments.

We initially evaluated the interaction of both ligands with nucleic acids by means of FRET melting experiments. The nucleic acid sequences, topology and genome localization are listed in Table S2 and cover parallel (cMyc, Kit1 and Kit2), antiparallel (HTelo‐Na, 22CTA and TBA), mixed (HTelo‐K and Bcl2), hybrid 1 (24TTG) and hybrid 2 (26TTA) conformations of G4 s. We included in the study one RNA G4 structure (F21T‐RNA) showing a parallel conformation, and a double‐stranded DNA (ds26) showing a B‐type duplex conformation, to assess the selectivity of the ligands for G4 vs. duplex DNA. The sequences termed HTelo, 26TTA and 24TTG are derived from telomeric regions, while cMyc, Kit1, Kit2 and Bcl2 are sequences from oncogene promoter regions. Interestingly, the pendant arms are key to stabilize the DNAs, being Spiro‐NMe3 (**1**) a strong stabilizing agent for both DNA and RNA G4s (Δ*T*_m_=10–35 °C at ratio 1 : 10), in contrast to Spiro‐SO3 (**2**), which lacks of any stabilization effect (Figure 2).

Of outmost importance is the negligible stabilization effect of Spiro‐NMe3 (**1**) for the duplex ds26, indicating selectivity for G‐quadruplex over duplex DNA. By taking into account the ratio 1 : 10, Spiro‐NMe3 (**1**) stabilizes largely the telomeric G4 HTelo both in potassium (Δ*T*_m_>35 °C, mixed topology) and sodium (Δ*T*_m_=24.8 °C, antiparallel topology) conditions as well as the G4s derived from Kit and Bcl oncogenes (Δ*T*_m_>35 °C for Kit1, 27.9 °C for Kit2 and 30.5 °C for Bcl2). Both hybrid conformations adopted by 24TTG and 26TTA G4s are less stabilized (Δ*T*_m_<12 °C) than other conformations. With regards to RNA, Spiro‐NMe3 (**1**) strongly stabilizes this G4 (Δ*T*_m_>26.5 °C for F21T‐RNA) indicating a large interaction for both DNA and RNA G4 structures. Strikingly, Spiro‐NMe3 (**1**) induces a moderate stabilization effect on the bitetrad G4 TBA at low molar ratio (Δ*T*_m_=19.4 °C at [L]:[DNA]=1 : 5).

To assess the G‐quadruplex versus duplex selectivity of Spiro‐NMe3 (**1**), we carried out competition FRET melting assays with F21T and Kit1 because of the larger stabilization effect observed on these G4s (Figure S11). The addition of 50 equivalents of calf thymus DNA (concentration in base pair) reduces the stabilization of G4 structures formed by F21T‐K/Na to a sixth of the Δ*T*_m_ values (from Δ*T*_m_ ≈30 °C to ≈5 °C, Figure S11) suggesting a moderate selectivity for G4 over duplex. With regards to Kit1, the stabilization decreases from 11.5 °C to 6.0 °C. These assays suggest a higher interaction of Spiro‐NMe3 (**1**) for G4 s and a moderate selectivity for G4 over duplex structures.

### Fluorescence emission assays

**Figure 3:**
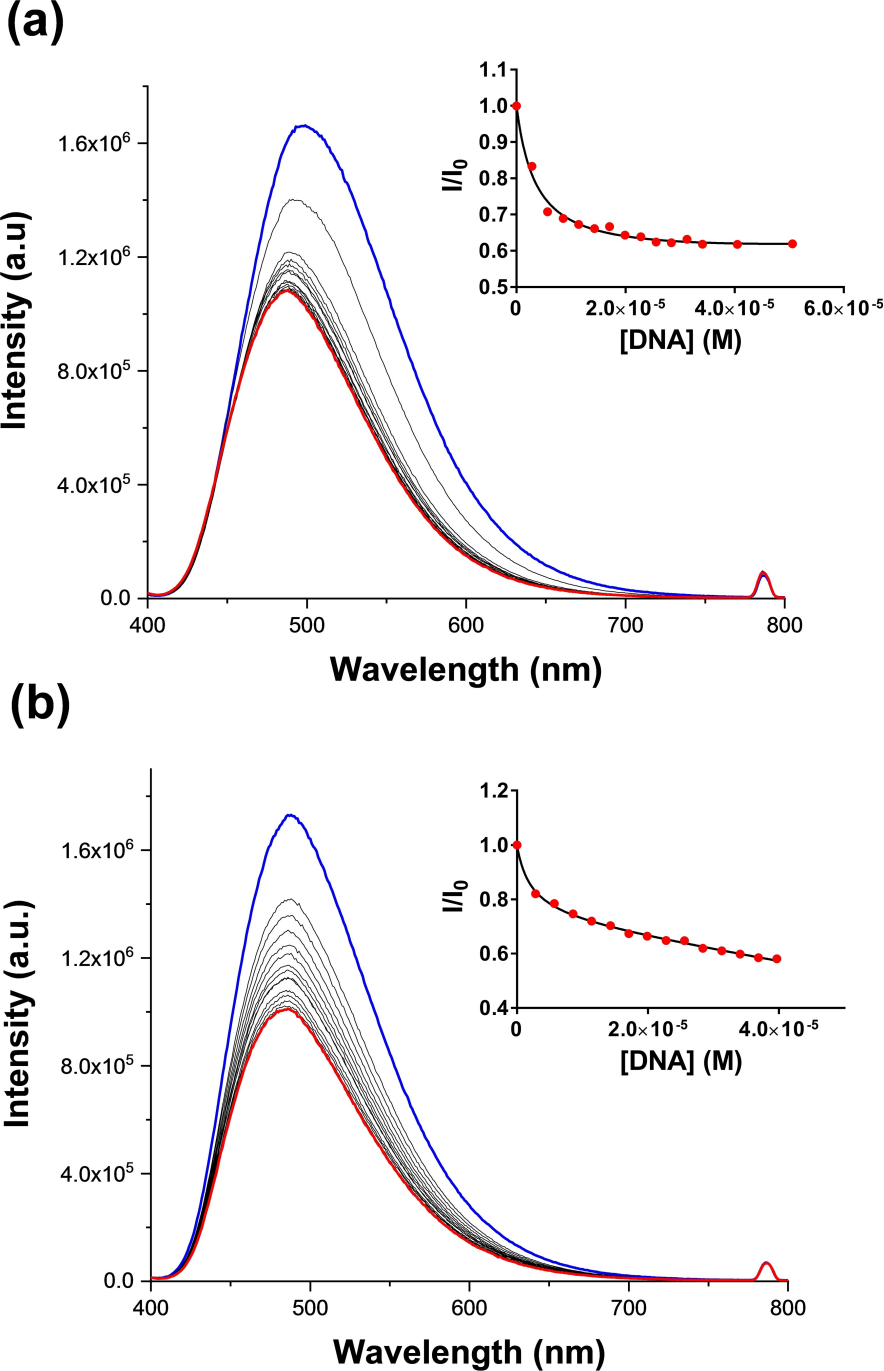
Fluorimetric titrations of (a) Spiro‐NMe3 (**1**) and (b) Spiro‐SO3 (**2**) with cMyc in Tris 10 mM, KCl 100 mM, pH=7.4, [Ligand]=3 μM. Insets: Plot of I/I_o_ *vs*. [DNA].

Having assessed the stabilization effect, we evaluated the fluorescence emission variation of the spirobifluorenes (**1** and **2**) upon interaction with different G‐quadruplex and duplex structures. We used the non‐labeled telomeric G4 HTelo and the promoter G4s from cMyc, Kit1 and Bcl2 (see Table S2). In addition, we included in the study two different polymeric duplexes of DNA, the calf thymus DNA and the ds26 with B‐type duplex conformation, and the polymeric RNA poly A ‐poly U with a A‐type duplex conformation. To assess the binding to proteins, we evaluated the binding to human serum albumin (HSA). Both spirobifluorenes show a fluorescence emission band upon excitation at 380 nm in buffered conditions (Tris 10 mM, KCl 100 mM, pH 7.4) centered at 505 nm (Figure 3). The bands of both ligands show a decrease in emission intensity upon addition of DNA and RNA although the variation depends on the nucleic acid structure (Figure 3 and Figures S12–S18).

The stability constant values (*K*_a_) were calculated from the titrations and are gathered in Table 1. For G4 s, the values are higher for the Spiro‐NMe3 ligand than the Spiro‐SO3, being one order of magnitude higher for the cationic spirobifluorene (**1**). Taking into account the identical central core of the spirobifluorene unit of the ligands, the stronger interaction shall be rationalized considering the net charge of the molecules. Ligand **1** can form electrostatic interactions between the tetraalkylammonium moieties and the negative charged backbone of the G‐quadruplexes, which cannot be formed by the sulfonate derivative **2**. Interestingly, the binding of the Spiro‐NMe3 (**1**) towards telomeric G4 (HTelo) is very high, suggesting a certain selectivity for this structure. With regards to the duplexes, Spiro‐NMe3 (**1**) shows high interaction to B‐type DNA (ctDNA and ds26) whereas no interaction was detected for RNA polymer with A‐type conformation. In contrast, Spiro‐SO3 (**2**) shows only a considerable binding towards calf thymus DNA. Finally, no binding was observed between Spiro‐NM3 (**1**) and the human serum albumin, while Spiro‐SO3 (**2**) shows a blue shifted and an enhancement of the emission as observed previously with bovine serum albumin (BSA),^[16]^ suggesting a similar interaction between the negative charged Spiro‐SO3 and HSA.

**Table 1:** Values of the stability constants (*K*_a_, M^−1^) obtained from the fluorimetric titrations of **1** and **2** with DNA, RNA and HSA.

	Spiro‐NMe3 (1)	Spiro‐SO3 (2)
HTelo	>10^7^	7.2(2)x10^4 [a]^
cMyc	4.5(0.8)x10^5^	5.6(0.1)x10^4^
Kit1	4.0(0.4)x10^5^	<10^3^
Bcl2	5.4(0.5)x10^5^	5.5(0.3)x10^4^
ds26	3.5(0.5)x10^5^	<10^3^
ctDNA	2.8(0.5)x10^5^	1.4(0.5)x10^5^
pAU	<10^3^	
HSA	<10^3^	<10^3^

[a] Values in parenthesis are standard deviation of at least three independent experiments.

### Fluorescent Intercalator Displacement (FID) assays

In order to support the findings from the FRET‐melting and fluorescence titrations results, Fluorescence Indicator Displacement (FID) assays were performed to investigate the binding of **1** and **2** towards G4s (HTelo, cMyc and kit1) and a duplex (ds26). The FID assays follow the decrease in the fluorescence emission of the thiazole orange (TO) upon the ligand‐induced displacement of TO from the DNA‐TO adduct. A value of the displacement effect is represented by the value of DC_50_ obtained from the plot of the percentage of displacement against the concentration of the ligand.^[19]^

**Figure 4:**
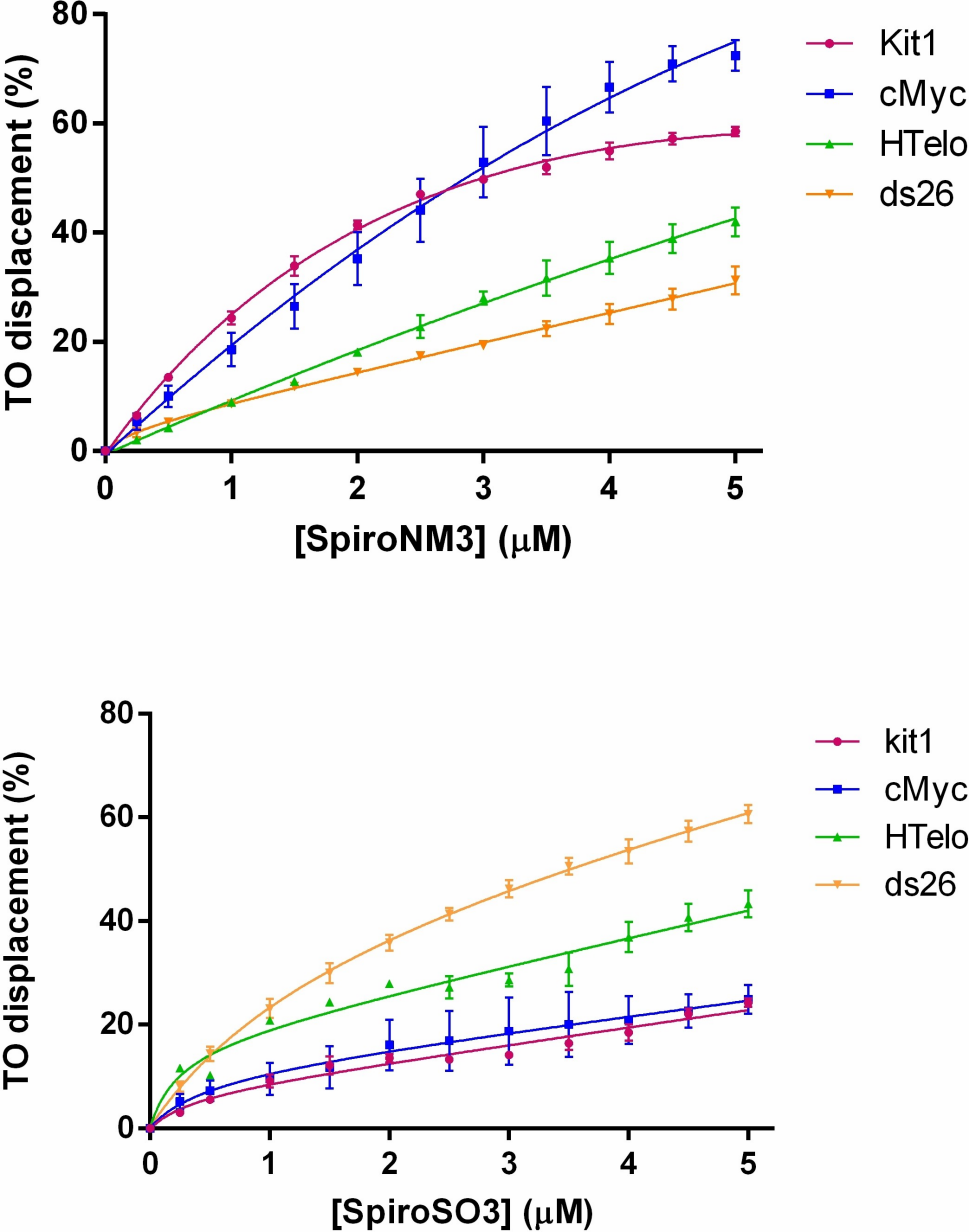
FID titration curves of DNA G4 (Kit1, cMyc and HTelo) and duplex ds26 with Spiro‐NMe3 (**1**, top panel) and Spiro‐SO3 (**2**, bottom panel).

Spiro‐NM3 (**1**) shows a larger displacement of TO from G4s than duplex, showing DC_50_ values of 2.84 and 3 μM for cMyc and Kit1 G4 structures (Table 2 and Figure 4) while TO is barely displaced from the duplex structure (ds26, Figure 4). On the other hand, Spiro‐SO3 (**2**) exhibits a stronger interaction for duplex as shown in Figure 4, being the duplex structure the only DNA with a DC_50_ value below 5 μM. These FID assays confirm the selectivity of the ligands towards the two different DNA structures mainly depending on the tetraalkylammonium or sulfonate moieties attached to the spirobifluorene scaffold.

**Table 2:** DC_50_ values (μM) calculated from the titration of spirobifluorenes to solutions of three different G4s (HTelo, cMyc and kit1) and duplex DNA (ds26). All values are average from three independent experiments with consistent results throughout.

	Spiro‐NMe3 (1)	Spiro‐SO3 (2)
HTelo	>5	>5
cMyc	2.84	>5
Kit1	3.00	>5
ds26	>5	3.52

### Molecular modelling of 1 with G4 and duplex structures

**Figure 5:**
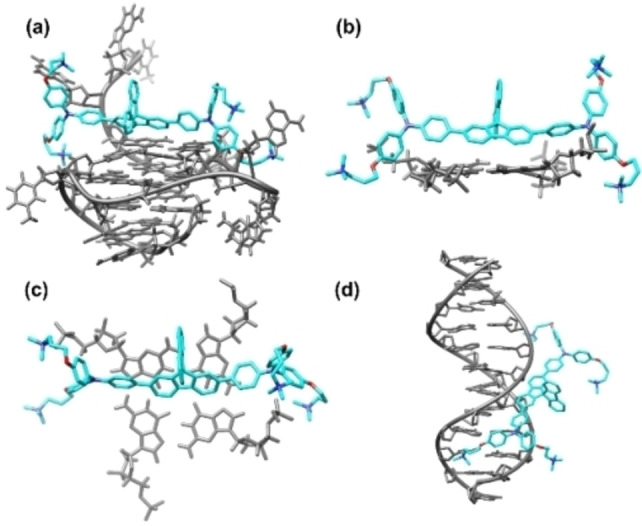
(a) Full structure of the Spiro‐NMe3 (**1**) with antiparallel G4 (PDB:2MGN). (b) Side view showing the positioning of the Spiro‐NMe3 (**1**) with the top G‐tetrad. (c) Simplified top view of the interactions between the Spiro‐NMe3 (**1**) and G4. (d) Full structure of the Spiro‐NMe3 (**1**) with duplex. G4 structure obtained from PDB: 2MGN and duplex from PDB: 296D.

Having established the interaction of Spiro‐NMe3 (**1**) for DNA structures, we were interested in gaining further insights into the binding mode of the ligand to explain the selectivity for G4s over duplex. Molecular docking studies were conducted using Autodock 4.2.^[20]^ The energy minimum conformers obtained from the docking of Spiro‐NMe3 (**1**) and the G‐quadruplex structure (see Materials and Methods for details) positioned the ligand on the top of the G‐quadruplex with the spirobifluorene unit bended with regards to the external G‐quartet (Figure 5 and Figures S19–S20), preventing efficient π–π interactions among these fragments. Nevertheless, one of the triphenylamine units is oriented towards the groove while the other triphenyl moiety is wrapping the adenine base formed in the loop. The conformer generates multiple binding contacts between the positive charged tetraalkylammonium groups and the phosphate backbone of the DNA structure (Figure S19). Then, we studied the double stranded DNA model by docking analysis. Spiro‐NMe3 (**1**) interacts through the minor groove of the B‐type duplex (Figure 5 and Figures S21–S22), in which one of the fluorene moieties is located externally to the groove and the pendant arms are pointing out the helical structure forming weak interactions (Figure S21). Overall, the docking studies point that the main driving forces of the interaction are the formed between the pendant arms while the rigid orthogonal spirobifluorene moiety has not the adequate binding pocket G4 and, in particular, for duplex structures, in contrast with ligands containing flat aromatic core such as naphthalene diimides.^[21]^

### Cellular localization studies

**Figure 6:**
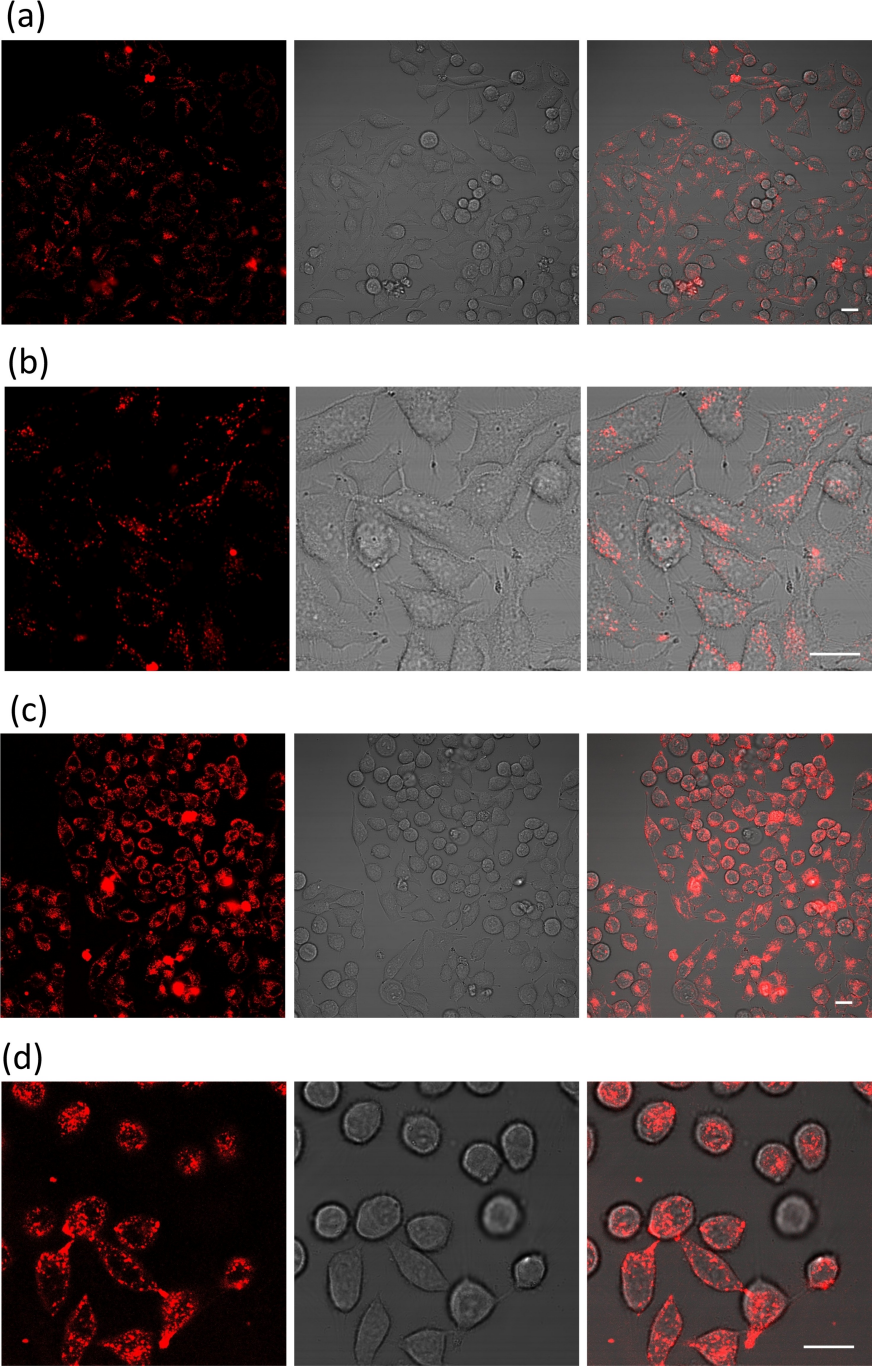
Confocal fluorescence images of HeLa cells incubated with Spiro‐NMe3 (a and b) and Spiro‐SO3 (c and d) (20 μM, 2 h). Left panel is the fluorescence emission of the ligands (λ_exc_=420 nm), central panel is bright field and right panel is the merged images from fluorescence and bright field. Bar, 20 μm.

Upon assessing the interaction with nucleic acids, we performed experiments using confocal fluorescence microscopy to assess the cell localization of ligands. Initially, we evaluated the cellular viability using the MTT assay in the HeLa cancer cell line and no toxicity was observed at 24 h even at the high concentration used of 100 μM (see Figure S23). Then, HeLa cells were incubated with the ligands during 2 h (20 μM) and visualized by fluorescence confocal microscopy (Figure 6). Both ligands show a clear cytoplasmic localization (see Figure 6), confirming the cellular uptake of the spirobifluorenes in this cell line.

**Figure 7:**
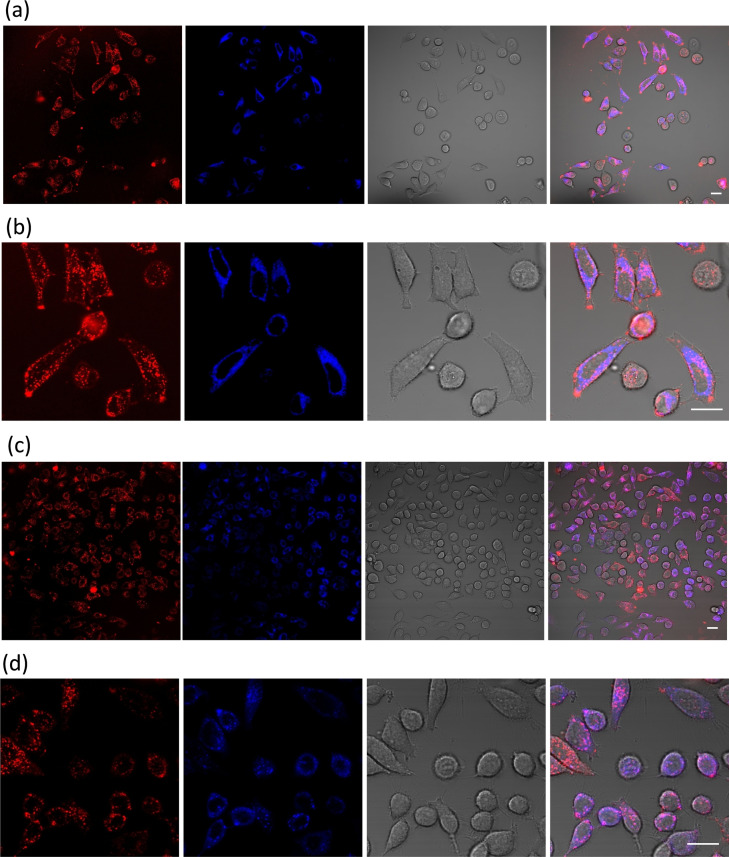
Confocal fluorescence images of HeLa cells incubated with Spiro‐NMe3 (20 μM, 2 h) and Mitotracker DR (a and b) or Lysotracker DR (c and d). Extreme left panel is the fluorescence emission of the ligands (λ_exc_=420 nm), central left panel is fluorescence emission of DR dyes (λ_exc_=650 nm), central right panel is bright field and extreme right panel is the merged images from fluorescence and bright field. Bar, 20 μm.

To further explore the cellular compartment accumulation of the ligands, we used co‐localization dyes, such as Mitotracker Deep Red (DR) and Lysotracker DR, to determine in which cytoplasmic organelle are accumulated the ligands. It is worth to note that the high aqueous solubility of the dyes allows carrying out the localization experiments without the addition of organic co‐solvents, like DMSO, thus preserving the cellular integrity. Both ligands show accumulation in the lysosomes rather than the mitochondria because of the larger overlapping of fluorescence emission between the spiro derivatives and the lysotracker dye (Figure 7, Figures S24–S26). The Pearson's Correlation Coefficients calculated from different images for Spiro‐NMe3 (**1**) and Spiro‐SO3 (**2**) in the lysosomes were 0.58 and 0.59 respectively, while the values for the ligands in the mitochondria were 0.43 and 0.32, confirming the lysosome targeting. The ability of both ligands to accumulate into lysosomes can attributed to the presence of two triphenylamine moieties that have been shown to target lysosomes.^[22]^ Nevertheless, both ligands show other localization in the cells that need to be further explored with other co‐localization dyes.

## Conclusion

In this work, we have synthesized and characterized two fluorescent spirobifluorene ligands bearing either positive (Spiro‐NMe3, **1**) or negative charge (Spiro‐SO3, **2**) attached to the same spirobifluorene core, which provide high solubility in water and buffered solutions. Spiro‐NMe3 contains four alkylammonium substituents while Spiro‐SO3 presents four sulfonate groups, resulting in a change of the overall net charge of the ligands from positive (+4) for **1** to negative (−4) for **2**. Here we demonstrated that this feature is pivotal on the binding towards different DNA structures. Spiro‐NMe3 interacts with G4s structures and shows preference for G4s over duplex by means of FRET melting, fluorescence and FID experiments, while Spiro‐SO3 exhibits higher binding affinity to duplex DNA than to G4s. This series of spirobifluorene molecules represents an example of DNA‐ligand interaction governed by the side arms of the ligands as well as the DNA conformation. The cell assays confirm the lower toxicity together with the internalization of both ligands, which showed higher accumulation in the lysosomes.

The moderate quenching of the fluorescence emission from both molecules upon DNA binding, suggest that they can be applied for imaging nucleic acids in cells, which we are currently exploring. These results open a novel scenario for the design of fluorescent molecules for selective DNA binding, which can impact in the field of detection assays and bioimaging.

## Experimental Section

**General**: All reagents were obtained from commercial sources and used without further purification, unless otherwise noted. All dry reactions were performed under argon atmosphere using glassware that was flamed under high‐vacuum and backfilled with argon. Organic solvents were dried by keeping them over molecular sieves 4 Å. Column chromatography was carried out on silica gel Si60, mesh size 0.040–0.063 mm (Merck, Darmstadt, Germany). Flash chromatography was carried out on silica gel mesh size 230–400 (J. T. Baker) and thin layer chromatography on aluminum sheets pre‐coated with silica gel 60 F254 (E. Merck). ^1^H NMR (400 MHz) and ^13^C NMR (101 MHz) spectra were obtained with a Bruker Neo 400 spectrometers. Chemical shifts (δ) are given as part per million (ppm) downfield from tetramethylsilane. The solvent signals of DMSO (^1^H: 2.5 ppm, ^13^C: 39.52 ppm) and CD_2_Cl_2_ (^1^H: 5.32 ppm, ^13^C: 53.84 ppm) chemical shifts were used as secondary chemical shift reference. The assignment of protons and carbon atoms was carried out by bidimensional NMR experiments (COSY, heterocorrelate ^1^H−^13^C) (see Supporting Information). The unlabeled and labeled DNA oligonucleotides were purchased from IDT DNA as HPLC grade and the labeling. dyes were 5’‐FAM and 3’‐TAMRA. Ligands were dissolved in miliQ water to give 5 mM stock solutions. All solutions were stored at ‐20 °C and defrosted and diluted immediately before use using suitable buffer to the appropriate concentrations.

### Synthesis

*Spiro‐Br* (**3**): A dried three‐neck flask equipped with condenser and magnetic stir bar was charged with **4** [16] (200 mg, 0.23 mmol, 1.0 equiv.), K_2_CO_3_ (anhydrous, 508 mg, 3.68 mmol, 16.0 equiv.) and KI (cat.). The mixture was stirred in acetone (dry, 20 mL) for 30 min. Afterwards, 1,3‐dibromopropane (1 mL, 9.81 mmol, 42.7 equiv.) was added and the reaction mixture was refluxed at 80 °C overnight. The reaction mixture was filtrated over celite and washed with EtOAc, acetone, MeOH and DCM. The solvents were evaporated and the crude product was purified by silica column chromatography (eluent, DCM/EtOAc=8/1). Molecular formula: C_73_H_62_Br_4_N_2_O_4_. Yield: 75 % (234 mg, 0.17 mmol). ^1^H NMR (400 MHz, CD_2_Cl_2_): δ=7.88 (t, *J*=7.9 Hz, 4H), 7.59 (d, *J*=8.0 Hz, 2H), 7.37 (t, *J*=7.5 Hz, 2H), 7.27–7.20 (m, 4H), 7.12 (t, *J*=7.5 Hz, 2H), 7.02–6.94 (m, 8H), 6.89–6.75 (m, 16H), 4.05 (t, *J*=5.6 Hz, 8H), 3.61 (t, *J*=5.5 Hz, 8H), 2.29 (q, *J*=5.6 Hz, 8H) ppm. ^13^C NMR (101 MHz, CD_2_Cl_2_): δ=155.44, 150.18, 149.24, 148.42, 142.26, 141.36, 140.70, 140.50, 133.11, 128.23, 128.14, 127.66, 126.81, 126.46, 124.32, 122.03, 121.13, 120.70, 120.50, 115.67, 66.46, 66.03, 32.83, 30.71 ppm. MALDI^+^‐MS: *m/z* calcd for C_73_H_62_N_2_O_4_Br_4_^+^: 1350.14; found=1350.15.

**Figure 8:**
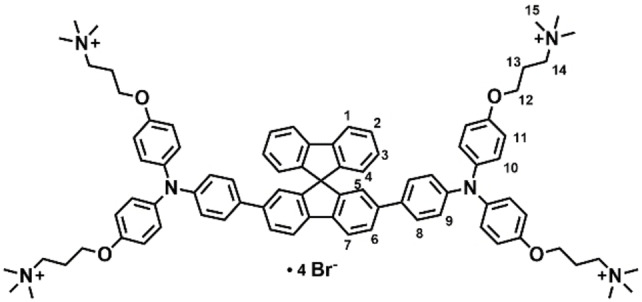
Assignment of atoms of Spiro‐NMe3 (**1**) for NMR analysis.

*Spiro‐NMe3* (**1**). In a Schlenk‐tube under Ar, *Spiro‐Br* (**3**) (57 mg, 0.042 mmol, 1.0 equiv.) was diluted in DMF (dry, 5 mL) and NMe_3_ (2 M in THF, 2.0 mL, 4.00 mmol, 100 equiv.) was added. The mixture was heated to 50 °C and stirred at that temperature overnight. The solvent and residual NMe_3_ were removed in vacuum to give the desired compound as light green solid. Molecular formula: C_85_H_98_Br_4_N_6_O_4_. Yield: 95 % (63 mg, 0.040 mmol). ^1^H NMR (400 MHz, DMSO‐*d*_6_): δ=8.08‐8.02 (m, 4H, H7,H1), 7.65 (d, *J*=7.5 Hz, 2H, H6), 7.41 (t, *J*=7.1 Hz, 2H, H2), 7.27 (d, *J*=8.0 Hz, 4H, H8), 7.15 (t, *J*=7.2 Hz, 2H, H3), 6.97–6.88 (m, 16H, H10, H11), 6.74–6.69 (m, 8H, H4,H5,H9), 4.00 (t, *J*=5.6 Hz, 8H, H12), 3.48 (t, *J*=8.0 Hz, 8H, H14), 3.10 (s, 36H, H15), 2.16 (br s, 8H, H13) ppm. ^13^C NMR (101 MHz, DMSO‐d_6_): δ=154.61, 149.31, 148.24, 147.62, 141.30, 140.29, 139.51, 131.56, 128.02 (C2,C3), 127.18 (C8), 126.40 (C10), 126.01 (C6), 123.58 (C5), 121.09 (C7), 120.62 (C1), 120.44 (C4), 120.13 (C9), 115.59 (C11), 65.54 (Cspiro), 64.98 (C12), 63.01 (C14), 52.29 (C15), 22.62 (C13) ppm (see Figure 8 for the labeling of 1).ESI^+^‐MS: *m/z* calcd for C_85_H_98_N_6_O_4_^4+^: 316.69069; found=316.69092; *m/z* calcd for C_85_H_98_BrN_6_O_4_^3+^: 449.22744; found=449.22694; *m/z* calcd for C_85_H_98_Br_2_N_6_O_4_^2+^: 713.30005; found=713.29951 (Figure 8).

**FRET melting assay**: Labeled DNA was dissolved as a 20 μM stock solution in MilliQ water, then annealed as a 400 nM concentration in potassium/sodium cacodylate buffer (pH 7.3) depending on the G4 at 95 °C for 5 min, and allowed to cool slowly to room temperature overnight. The buffer used for the antiparallel G4 HTelo Na was 100 mM NaCl, 10 mM LiCac, for the rest of G4s and duplex was 100 mM KCl, 10 mM LiCac. Ligands were dissolved from stock solutions (see above) to final concentrations in the buffer. Each well of a 96‐ well plate (Applied Biosystem) was prepared with 60 μL, with a final 200 nM DNA concentration and increasing concentration of tested ligands (0–4 μM). Measurements were performed on a PCR AriaMx (Agilent Technologies) with excitation at 450–495 nm and detection at 515–545 nm. Readings were taken from 25 °C to 95 °C at interval of 0.5 °C maintaining a constant temperature for 30 seconds before each reading. Each measurement was done in triplicate. The normalized fluorescence signal was plotted against the compound concentration and the Δ*T*_m_ values were determined.

**FRET competition assay**: Labelled oligonucleotides were annealed as a 400 nM concentration in potassium cacodylate buffer (10 mM KCl, 90 mM LiCl, 10 mM LiCac pH 7.3 for F21T‐K/Na and kit1) at 95 °C for 5 min, and allowed to cool slowly to room temperature overnight. Ligands were dissolved in stock solutions to final concentrations in the buffer. Each well of a 96‐well plate was prepared with a final 200 nM oligo concentration, 2 μM ligand concentration and ctDNA concentration to test (0 to 500 μM). Measurements were performed under the same conditions as those used in the FRET melting assay.

**Fluorescence emission titrations**: The DNA was dissolved in Tris buffer (100 mM KCl, 10 mM Tris pH 7.4) and annealed at 95 °C for 10 min before cooling to room temperature overnight. The concentration of DNA was checked using the molar extinction coefficients provided by the manufacturer. Annealing concentrations were approximately 500 μM. For emission titrations, ligands (5 μM) in the same buffer were titrated with the corresponding DNA until saturation of fluorescence. The emission spectra were recorded between 390 and 680 nm with an excitation wavelength of 380 nm in 1 cm path‐length quartz cuvettes. The emission spectra were recorded on a Varian Cary Eclipse Spectrometer. Spectra were smoothed using the Savitzky‐Golay algorithm and emission maxima were fitted to 1 : 1 binding model using the Levenberg‐ Marquardt algorithm and equations previously reported.^[23]^

**Fluorescent intercalator displacement (FID) assay**: Measurements were performed on a Varian Cary Eclipse Spectrometer following the protocol reported by Teulade‐Fichou's team.^[19]^ Oligonucleotides (quadruplexes and duplex) were prepared by heating the corresponding oligonucleotides at 90 °C in LiCaco buffer (100 mM KCl, 10 mM LiCaco pH 7.2) then slowly cooling to room temperature overnight. Oligonucleotide structures were formed at 250 μM strand concentration. The test is designed as follows: onto a mixture of prefolded quadruplex or duplex (1 μM) and TO (2 μM for G4s and 3 μM for ds26), in LiCaco buffer (100 mM KCl, 10 mM LiCaco pH 7.2), addition of increasing amount of ligand (from 0.25 to 5 equiv.) is followed by a 2 min equilibration period before the fluorescence spectrum is recorded. The fluorescence area (FA, 510–850 nm), converted in percentage displacement (PD, with PD=100–[(FA/FA_0_) ⋅ 100], FA_0_ being FA before addition of ligand), is then plotted versus the concentration of added ligand. To quantify the affinity of the ligands to different DNAs, DC_50_ values (i. e., the concentration needed to displace 50 % of TO from the DNA) were calculated and compared for the different compounds and DNAs used.

**Molecular docking studies**: Molecular docking studies were performed using Autodock 4.2.6 with the Lamarckian genetic algorithm. The ligand structure was minimized in Avogadro at the GAFF Force Field and then docked with G‐quadruplex (PDB: 2MGN) and duplex (PDB: 296D). In each case the structures were stripped of any existing counteranions, water molecules or ligands using Chimera 1.16. The structures were then imported into AutoDockTools‐1.5.6 and hydrogen atoms were added. A grid box encompassing the entire quadruplex and duplex was used in order to blind docking to be carried out. In each case, the lowest energy solution was taken. The docked structures were visualized and hydrogen bond distances measured using Chimera.

**Cell culture**: HeLa cells were grown in low glucose phenol red‐free Dulbecco's modified Eagle medium supplemented with 10 % fetal bovine serum and penicillin‐streptomycin at 37 °C with 10 % CO_2_ in humidified air. Cells were kept continuously under confluence before split twice a week and the possibility of contamination was excluded by regularly performing mycoplasma tests.

**Cell Viability (MTT) Assay**: The cytotoxic effects of ligands toward HeLa cells were assessed by MTT assay for cell viability. The cells were seeded at a density of 5×10^4^ cells/mL (if we consider 5000 cells in 100 μL of culture media per well in 96‐well plates). The culture medium was removed after the cells adhered to the wall, and they were treated with ligands at serial concentrations for 24 h. Then, the medium was removed, and the cells were washed with PBS. Finally, 90 μL of serum‐free without red‐phenol culture media and 10 μL MTT solution (5 mg/mL) were added to each well. After incubation for 4 h, the supernatant was removed and 100 μL DMSO was added to each well. The trays were then vigorously shaken to solubilize the formazan product and the absorbance at a wavelength of 490 nm was read on a microplate reader (MTX Labsystems, Vantaa, Finland) and analyzed. All MTT assays were performed three times in duplicate. A negative control was also performed by exposing cells only to culture medium and a positive control was conducted by using 0.1 % Triton X‐100.

**Cellular imaging**: Cells were seeded on chambered coverglass (*ca*. 2×10^4^, 300 μL, 0.8 cm^2^) for 6–24 h, then the media was replaced with fresh media phenol red‐free containing Spiro‐NM3 (**1**) or Spiro‐SO3 (**2**) (20 μM, 300 μL) for 2 h. Prior to imaging, the cells were washed with PBS and replaced with fresh growth media. Cells were imaged using a confocal fluorescence microscope (TCS SP5 Confocal, Leica Microsystems GmbH, Germany). Using a 63x magnification microscope objective (water immersion, NA 1/4 1.2) and an excitation wavelength of 420 nm for Spiro derivatives, images of the cells were recorded in both transmission and fluorescence modes. For the fluorescence images, the detection band was 430–500 nm which covered the emission range of the ligands. For co‐localization experiments, cells were washed with PBS and then incubated with Mitotracker Deep Red in PBS (400 nM, 300 μL, 15 min) or Lysotracker Deep Red in PBS (50 nM, 300 μL, 30 min). Mitotracker and Lysotracker Deep Red dyes were excited at 640 nm and the emission collected from 660–750 nm. Cells were then washed with PBS and fresh media added before imaging. ImageJ software was used to calculate the Pearson's correlation coefficients for the co‐localization of the ligands and the corresponding organelle‐targeting dyes.^[24]^

## Conflict of interest

The authors declare no conflict of interest.

1

## Supporting information

As a service to our authors and readers, this journal provides supporting information supplied by the authors. Such materials are peer reviewed and may be re‐organized for online delivery, but are not copy‐edited or typeset. Technical support issues arising from supporting information (other than missing files) should be addressed to the authors.

Supporting Information

## Data Availability

The data that support the findings of this study are available from the corresponding author upon reasonable request.
