# Supplementary material for: Modulating the G‐Quadruplex and Duplex DNA Binding by Controlling the Charge of Fluorescent Molecules
Source: Chemistry. 2022 Dec 8;29(6):e202203094. doi: 10.1002/chem.202203094 (PMC10107164; doi:10.1002/chem.202203094)
Supplement: Supplementary file 1 — Supporting Information [file CHEM-29-0-s001.pdf]

Chemistry–A European Journal

Supporting Information

Modulating the G-Quadruplex and Duplex DNA Binding by Controlling the Charge of Fluorescent Molecules

Ariadna Gil-Martínez, Sònia López-Molina, Cristina Galiana-Roselló, Andrea Lázaro-Gómez, Friederike Schlüter, Fabio Rizzo,* and Jorge González-García*

General Information

All reagents from commercial sources were used without further purification, unless otherwise noted. All dry reactions were performed under Ar atmosphere using glassware that was flamed under high-vacuum and backfilled with Ar. Organic solvents were dried by keeping them over molecular sieves 4A. Column chromatography was carried out on silica gel Si60, mesh size 0.040-0.063 mm (Merck, Darmstadt, Germany). Flash chromatography was carried out on silica gel mesh size 230-400 (J. T. Baker) and thin layer chromatography on aluminum sheets pre-coated with silica gel 60 F254 (E. Merck). ^{1}H NMR (400 MHz) and ^{13}C NMR (101 MHz) spectra were obtained with a Bruker Neo 400 spectrometers. Chemical shifts (δ) are given as part per million (ppm) downfield from tetramethylsilane. The solvent signals of DMSO (^{1}H: 2.5 ppm, ^{13}C: 39.52 ppm) and CD_2Cl_2 (^{1}H: 5.32 ppm, ^{13}C: 53.84 ppm) chemical shifts were used as secondary chemical shift reference. The assignment of protons and carbon atoms was carried out by bidimensional NMR experiments (COSY, heterocorrelate ^{1}H-^{13}C). The mass analyses have been carried out by using an Autoflex Speed MALDI-TOF (Bruker Daltonics) and an Orbitrap Velos Pro (Thermo Fisher Scientific). UV–Vis measurements were performed on a Jasco V-750 double-beam spectrophotometer and baseline corrected. Extinction molar coefficients were calculated by interpolation of data obtained from five solutions. Steady-state emission and excitation spectra were recorded on an FLS920 (Edinburgh Instruments) fluorescence spectrometer equipped with a single grating monochromator in both the excitation and the emission sides, and coupled to an R928P Hamamatsu photomultiplier; a 450W Xe arc lamp was used as the excitation source. The emission spectra were corrected for detection and optical spectral response of the fluorescence spectrometer through a calibration curve supplied by the manufacturer.

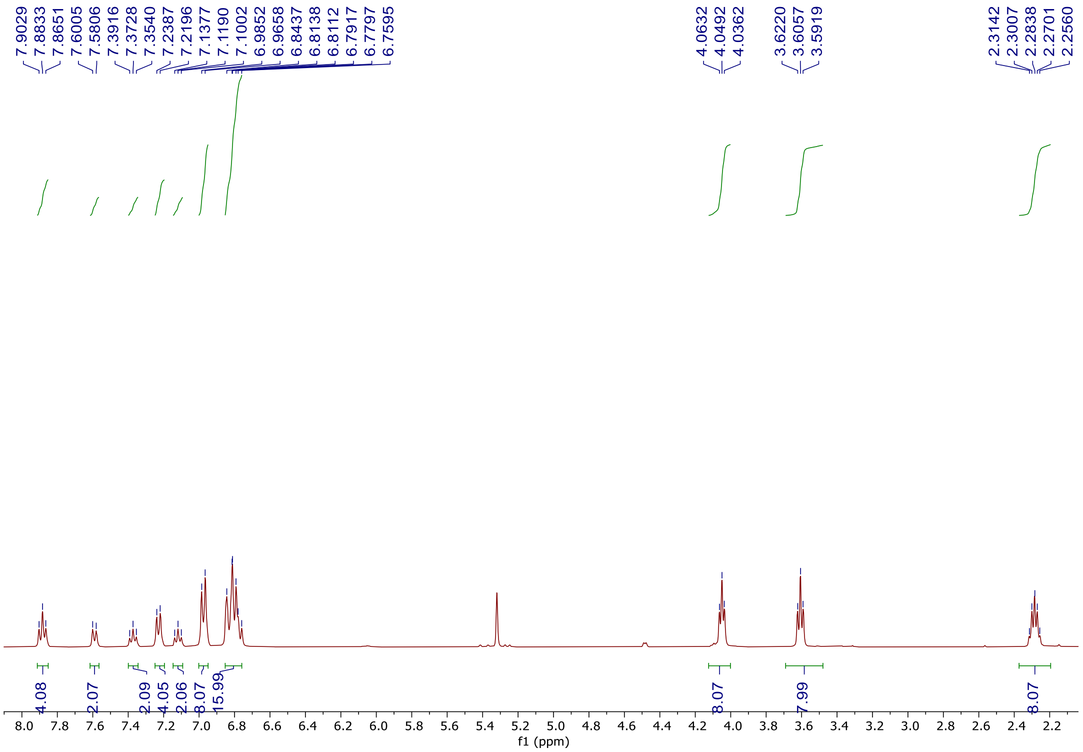

Figure S1. ^{1}H NMR spectrum of Spiro-Br (**3**) in CD_2Cl_2.

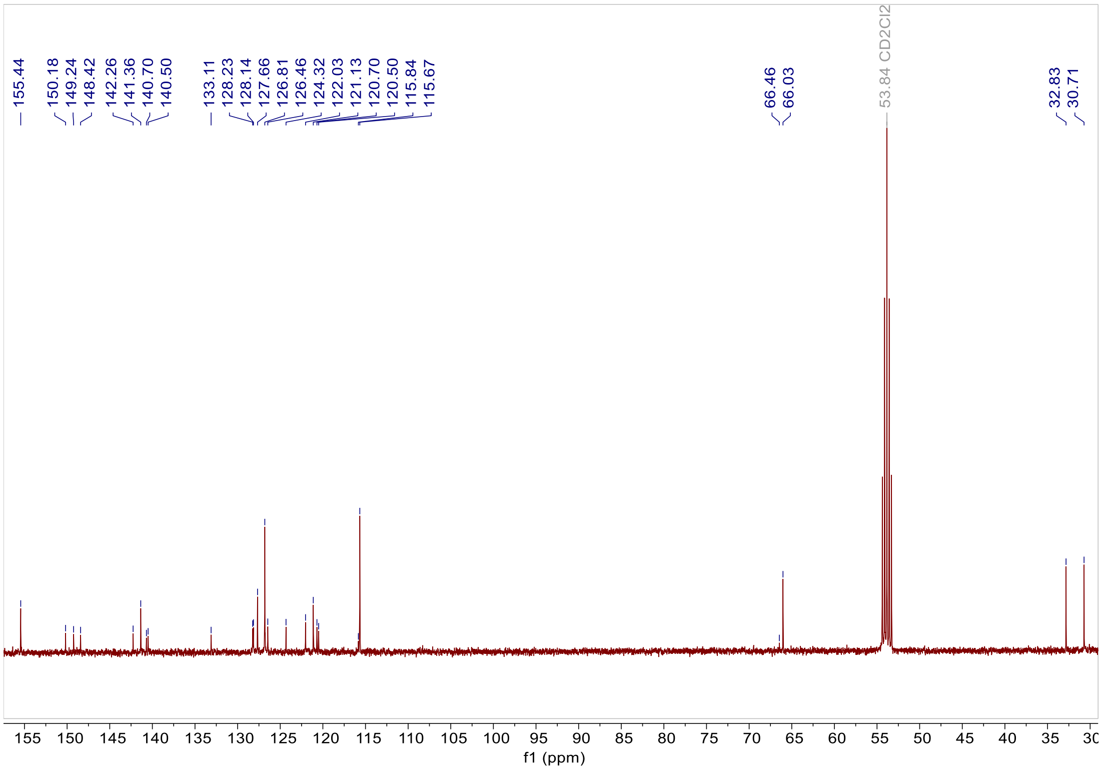

Figure S2. ^{13}C NMR spectrum of Spiro-Br (**3**) in CD_2Cl_2.

AutoFlex Speed (MALDI TOF)

Analysis Info

Analysis Name	Compound 3
Comment	Matrix = DCTB (CHCl3)

Acquisition method name	D:\Methods\flexControlMethods\RP_400-4000x_Da.par
Number of shots	1000
Position	E23
MSMS parent mass	
positive voltage polarity	POS

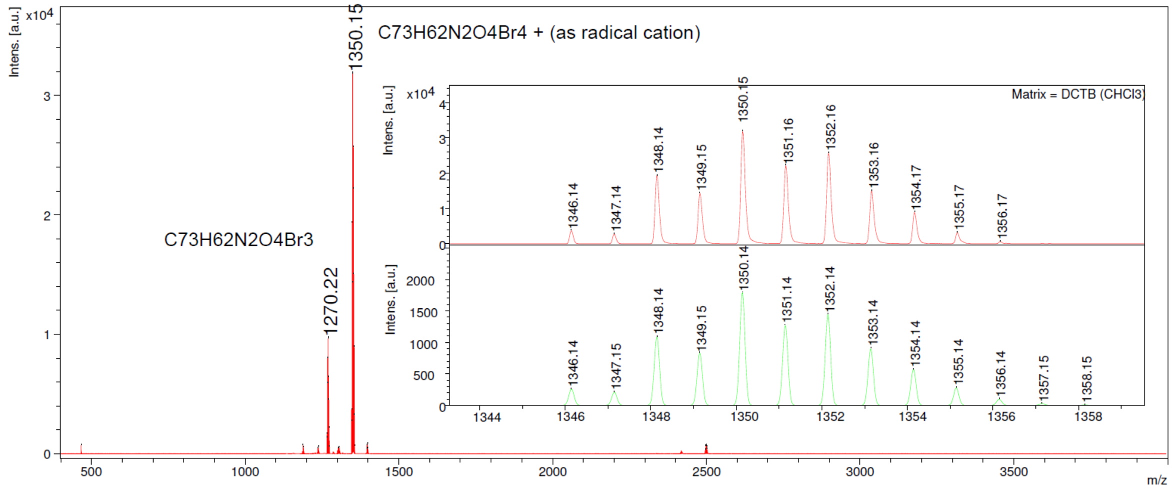

Figure S3. Mass spectrum of Spiro-Br (**3**). The inset indicates the calculated (green line, bottom) and measured (red line, top) molecular peak.

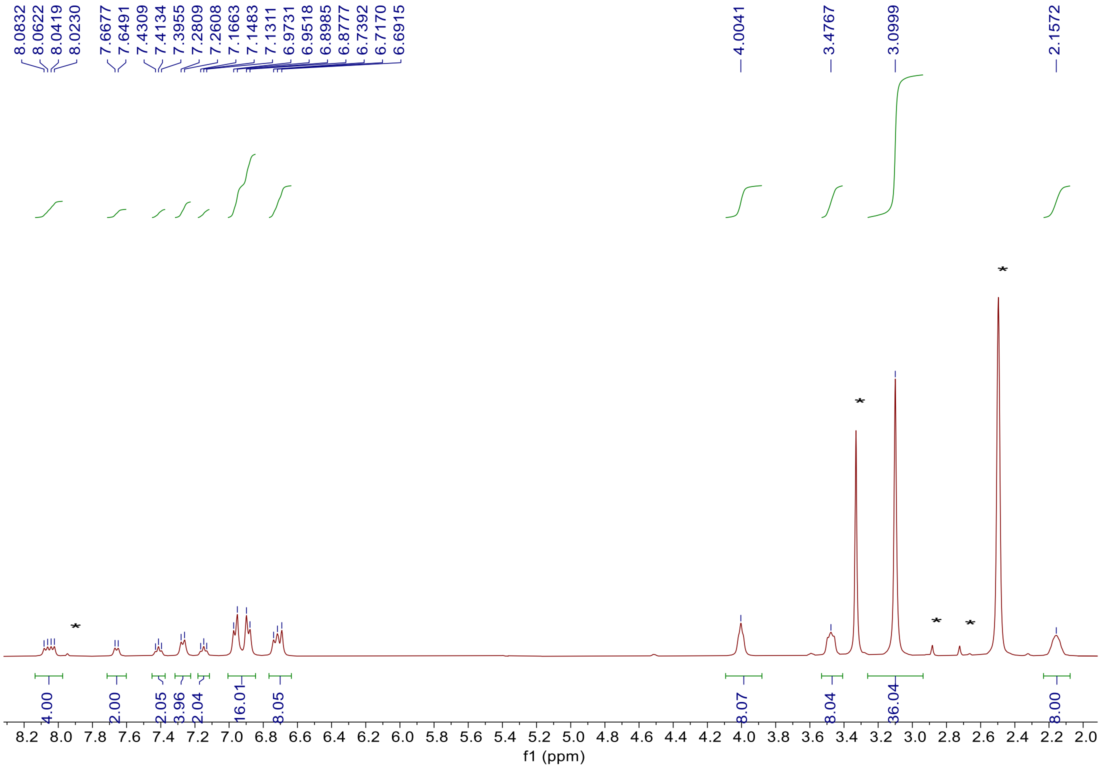

Figure S4. ^{1}H NMR spectrum of Spiro-NMe3 (**1**) in DMSO-d6. * indicates solvent peaks.

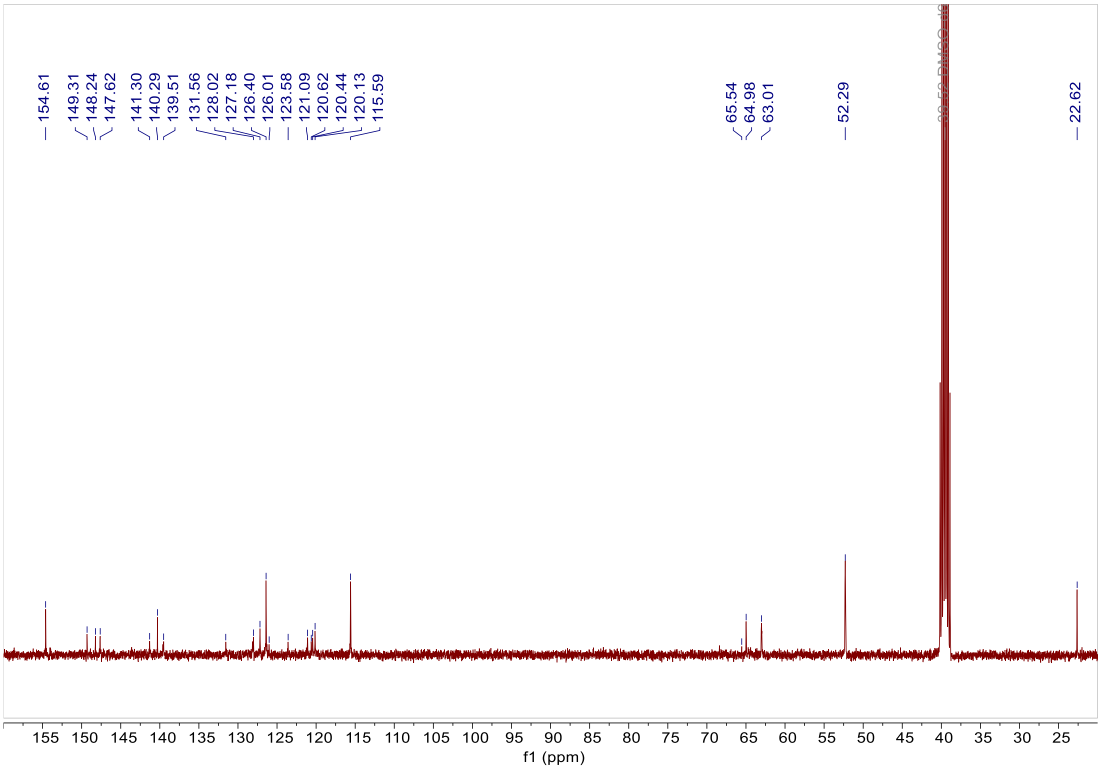

Figure S5. ^{13}C NMR spectrum of Spiro-NMe3 (**1**) in DMSO-d6.

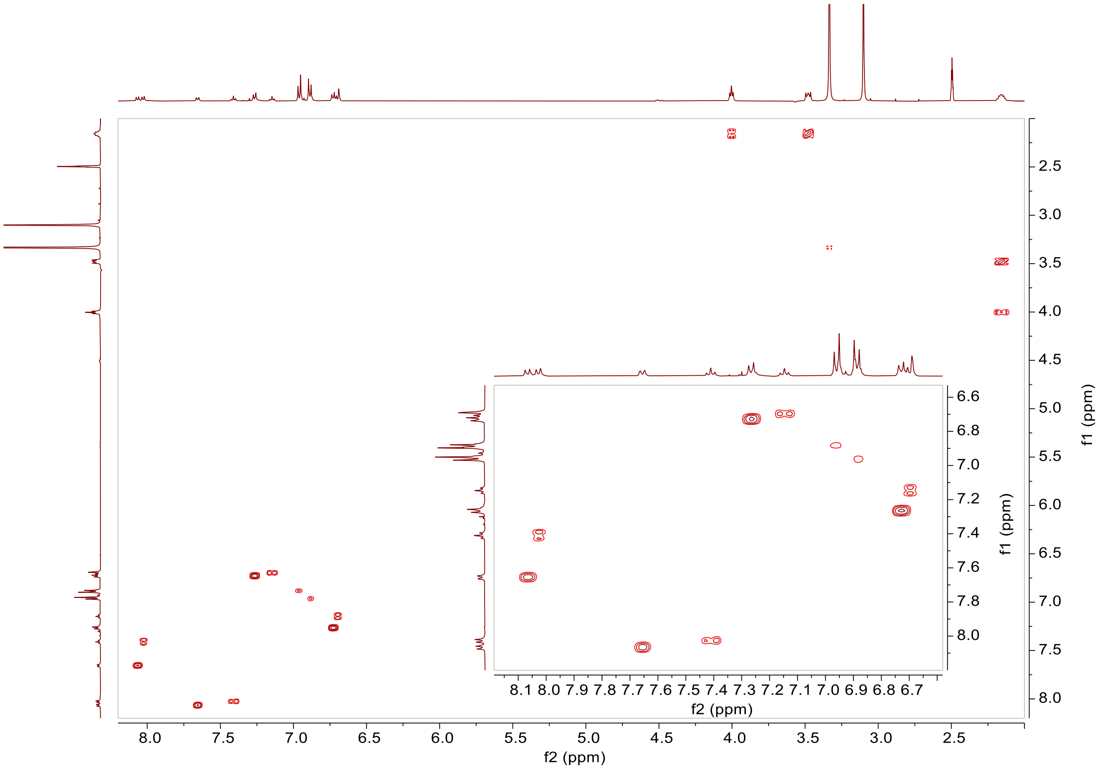

Figure S6. Bidimensional COSY NMR spectrum of Spiro-NMe3 (**1**) in DMSO-d6.

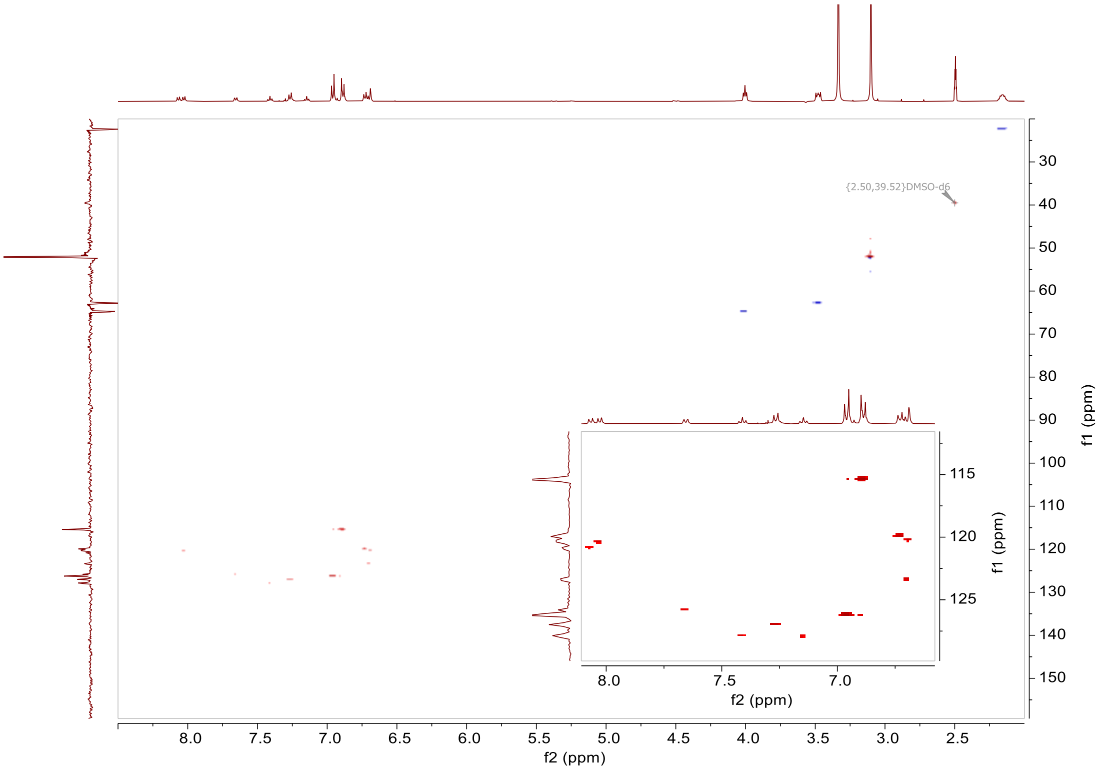

Figure S7. Bidimensional ^{1}H-^{13}C heterocorrelate NMR spectrum of Spiro-NMe3 (**1**) in DMSO-d6.

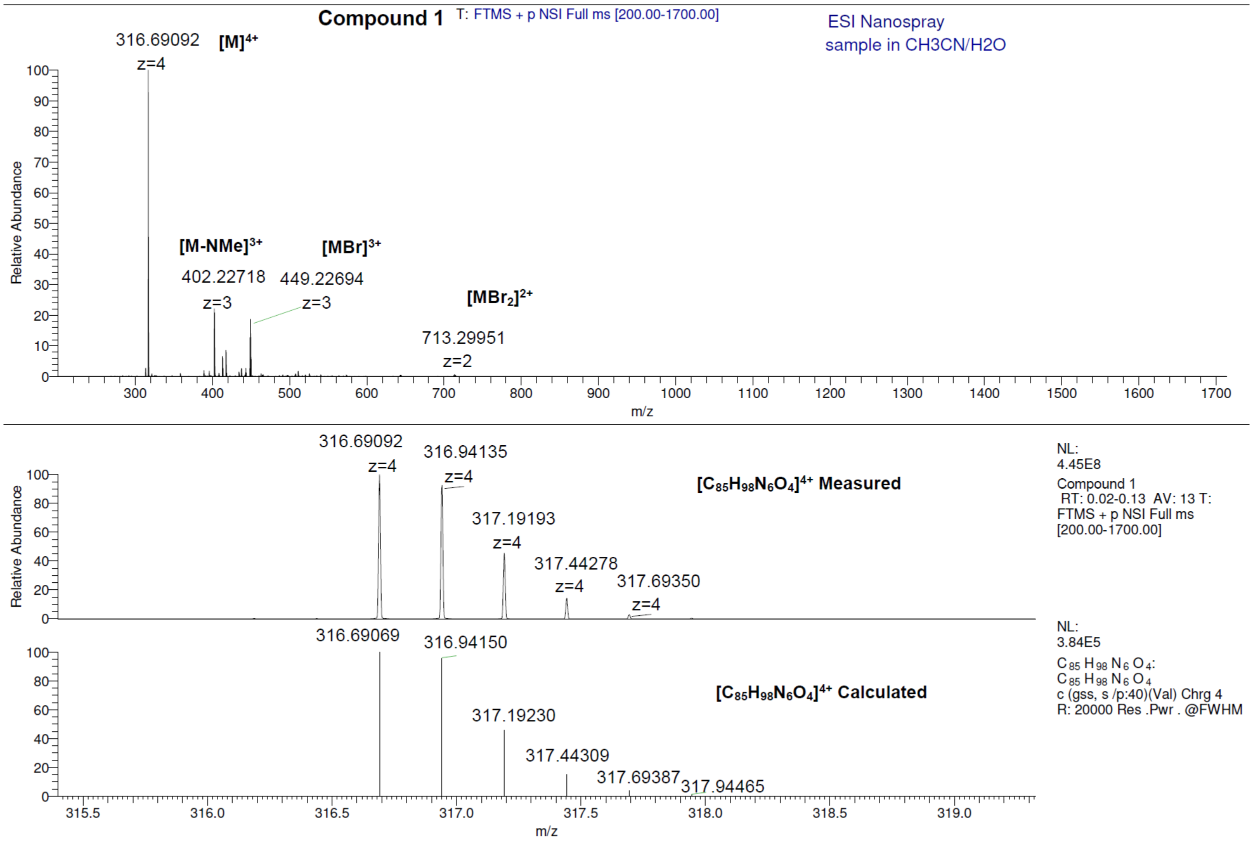

Figure S8. Mass spectrum of Spiro-NMe3 (**1**).

Table S1. Photophysical data of the two chromophores in different solvents.

Compound[a]	λ_{abs} (nm)	ε (cm^{-1} M^{-1})	λ_{em} (nm)	Stokes shift (cm^{-1})
**1 in DMF**	384	53270	508	6360
**1 in H_2O**	380	49260	505	6510
**1 in PBS**	381	28200	507	6520
**2 in DMF**[b]	385	63100	505	6170
**2 in H_2O**[b]	378	51290	480	5620
**2 in PBS**[b]	378	51290	480	5620

a: concentration used 3 µM. *b*: data from *Chem. Commun.*, **2018**, *54*, 642

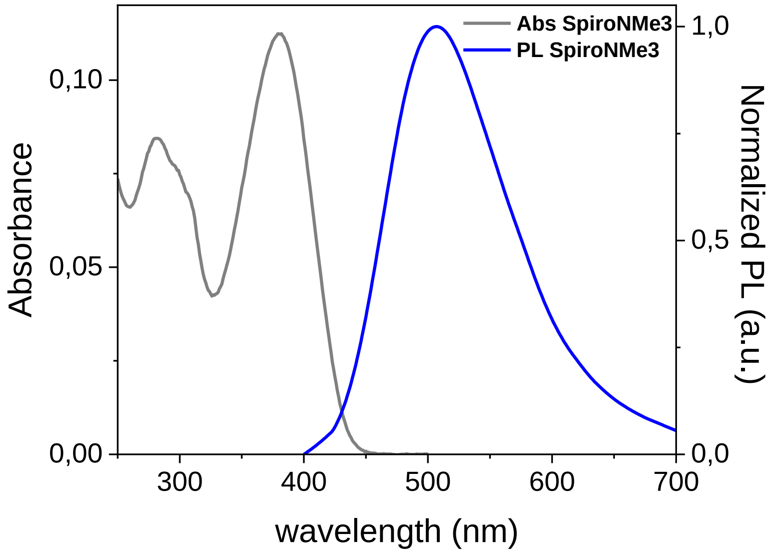

Figure S9. Absorption and emission spectra of Spiro-NMe3 (**1**) in H_2O (3 µM).

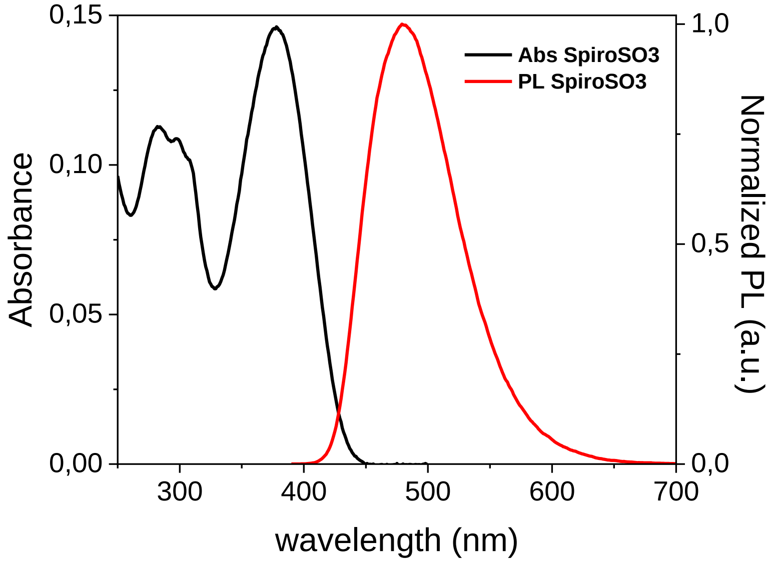

Figure S10. Absorption and emission spectra of Spiro-SO3 (**2**) in PBS (3 µM).

Table S2. List of DNA sequences used in this work, acronym, localization in the genome

Abreviation	Sequence (5’ → 3’)	Topology	Localization
**F21T-K**	GGGTTAGGGTTAGGGTTAGGG	Mixed	telomere
**F21T-Na**	GGGTTAGGGTTAGGGTTAGGG	Antiparallel	telomere
**cMyc**	TGAGGGTGGGTAGGGTGGGTAA	Parallel	promoter of cMyc
**22CTA**	AGGGCTAGGGCTAGGGCTAGGG	Antiparallel	telomere
**26TTA**	TTAGGGTTAGGGTTAGGGTTAGGGTT	Hybrid 2	telomere
**24TTG**	TTGGGTTAGGGTTAGGGTTAGGGA	Hybrid 1	telomere
**Kit1**	AGGGAGGGCGCTGGGAGGAGGG	Parallel	promoter of kit
**Kit2**	CGGGCGGGCGCGAGGGAGGGG	Parallel	promoter of kit
**Bcl2**	GGGCGCGGGAGGAATTGGGCGGG	Hybrid	promoter of Bcl2
**G4C2**	GGGGCCGGGGCCGGGGGCCGGGG		promoter of C9orf72
**TBA**	GGTTGGTGTGGTTGG	Antiparallel	Thrombin binding aptamer
**F21T-RNA**	r(GGGTTAGGGTTAGGGTTAGGG)	parallel	RNA
**ds26**	CAATCGGATCGAATTCGATCCGATTG	B-type duplex	
**HTelo22**	AGGGTTAGGGTTAGGGTTAGGG	Mixed	telomere

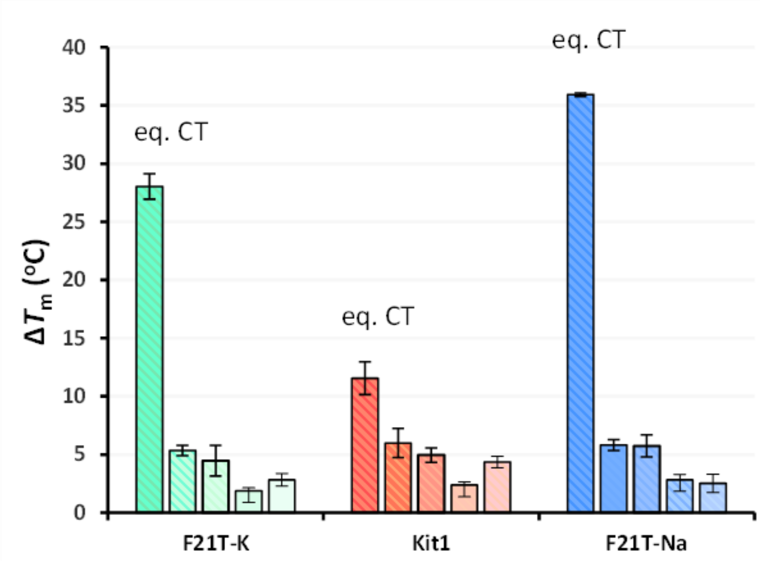

Figure S11. Representation of the competition FRET melting assays data for Spiro-NMe3 (**1**) (1.6 µM) with different labelled G4 sequences (0.2 µM) with increasing concentration of non-labelled calf thymus DNA as competitor (0, 10, 20, 50 and 100 µM). Errors denote the standard deviations of at least three independent experiments.

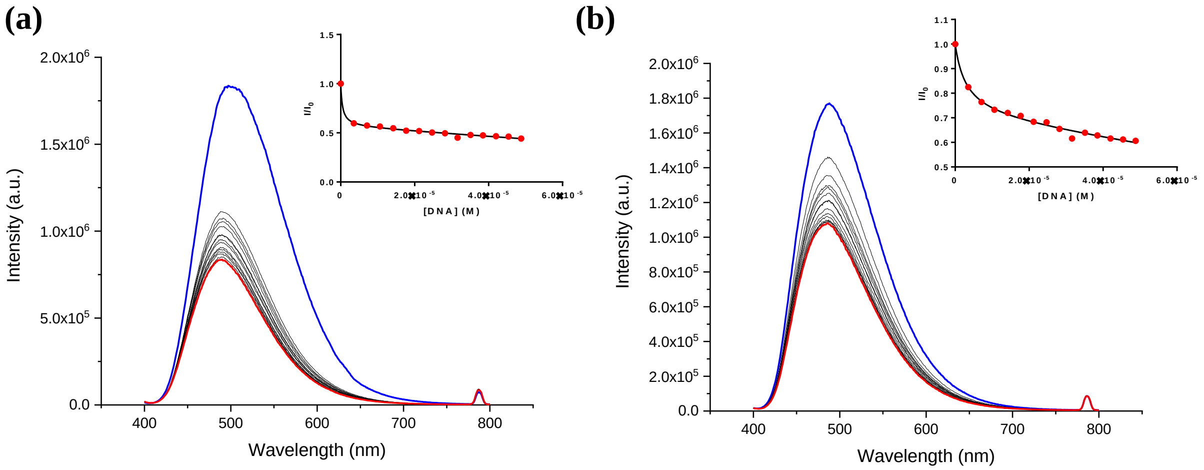

Figure S12. Fluorimetric titrations of (a) Spiro-NMe3 (**1**) and (b) Spiro-SO3 (**2**) with Htelo22 in Tris 10 mM, KCl 100 mM, pH= 7.4, [Ligand] = 3 µM. Insets: Plot of I/I_o *vs.* [DNA].

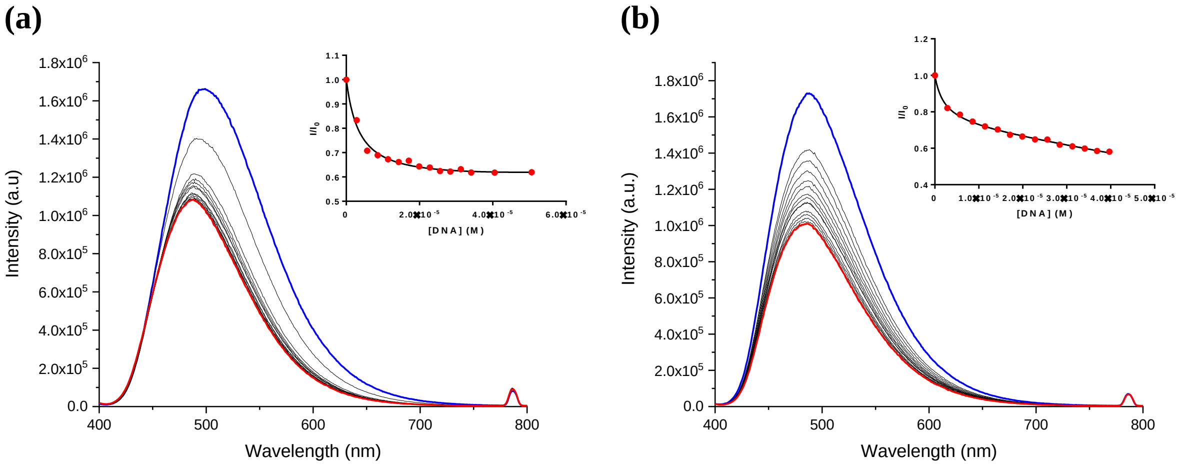

Figure S13. Fluorimetric titrations of (a) Spiro-NMe3 (**1**) and (b) Spiro-SO3 (**2**) with cMyc in Tris 10 mM, KCl 100 mM, pH= 7.4, [Ligand] = 3 μM. Insets: Plot of I/I_0 *vs.* [DNA].

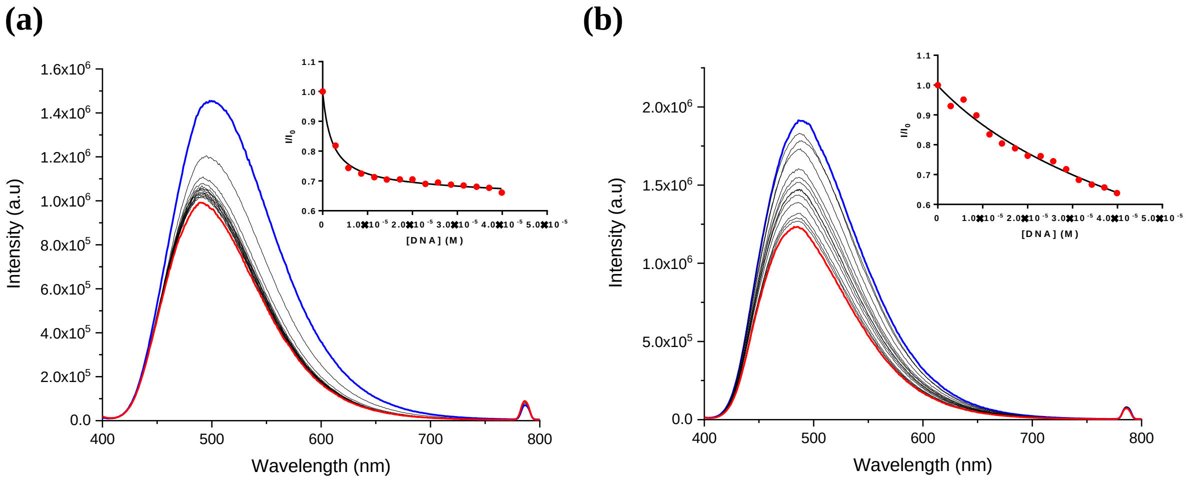

Figure S14. Fluorimetric titrations of (a) Spiro-NMe3 (**1**) and (b) Spiro-SO3 (**2**) with Kit1 in Tris 10 mM, KCl 100 mM, pH= 7.4, [Ligand] = 3 μM. Insets: Plot of I/I_0 *vs.* [DNA].

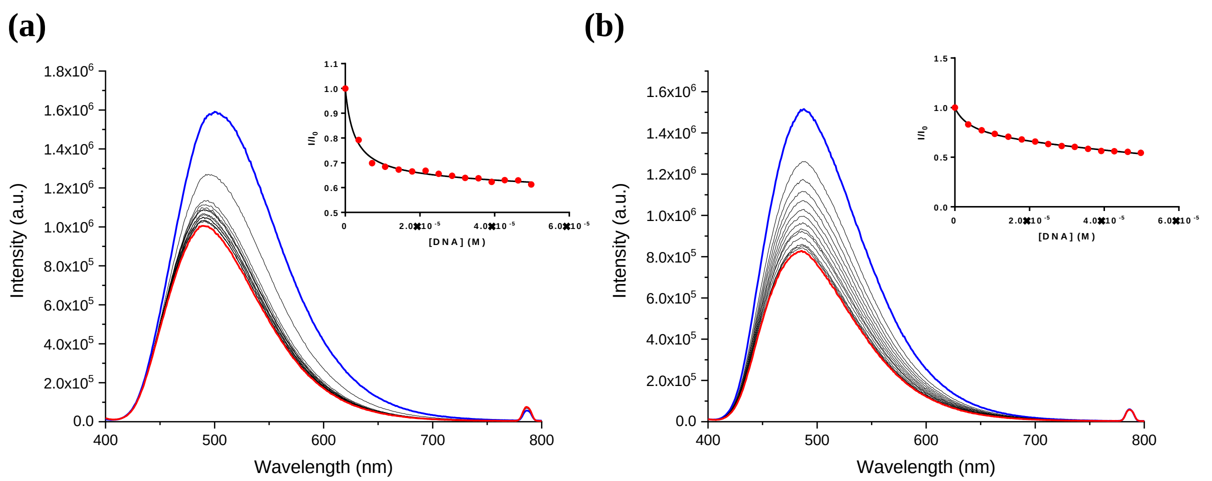

Figure S15. Fluorimetric titrations of (a) Spiro-NMe3 (**1**) and (b) Spiro-SO3 (**2**) with Bcl2 in Tris 10 mM, KCl 100 mM, pH= 7.4, [Ligand] = 3 µM. Insets: Plot of I/I_o *vs.* [DNA].

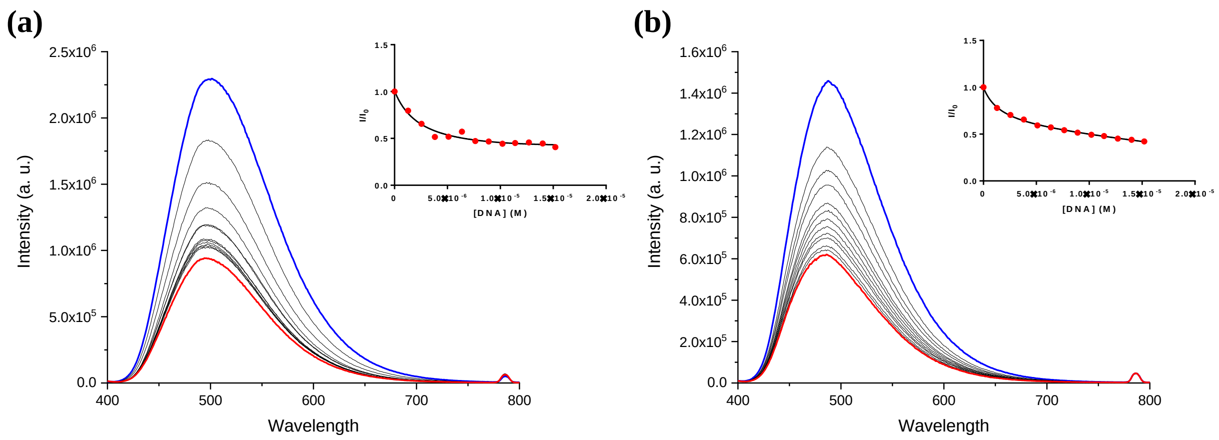

Figure S16. Fluorimetric titrations of (a) Spiro-NMe3 (**1**) and (b) Spiro-SO3 (**2**) with ctDNA in Tris 10 mM, KCl 100 mM, pH= 7.4, [Ligand] = 3 µM. Insets: Plot of I/I_o *vs.* [DNA].

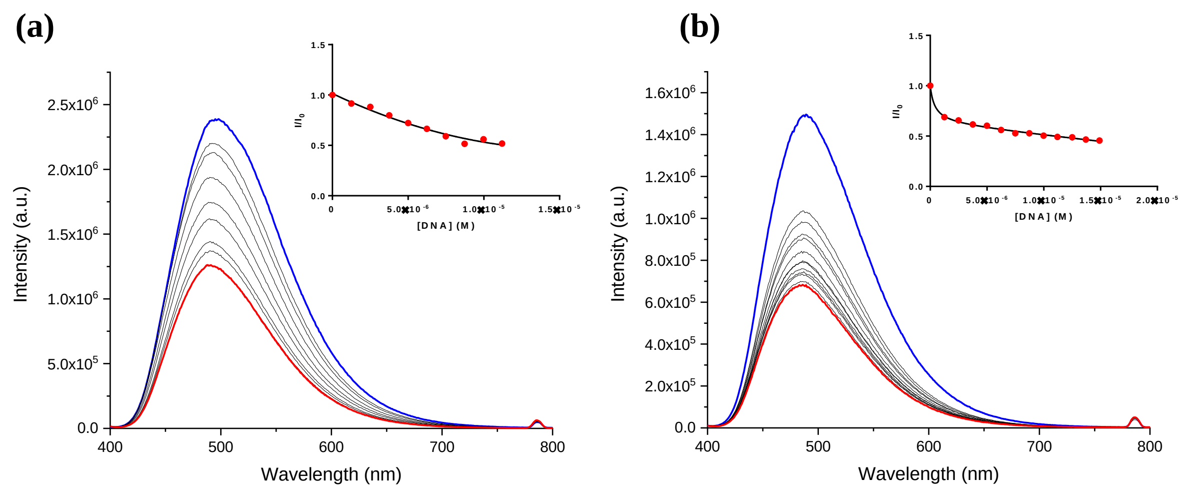

Figure S17. Fluorimetric titrations of (a) Spiro-NMe3 (**1**) and (b) Spiro-SO3 (**2**) with polyA-polyU in Tris 10 mM, KCl 100 mM, pH= 7.4, [Ligand] = 3 μM. Insets: Plot of I/I_0 *vs.* [DNA].

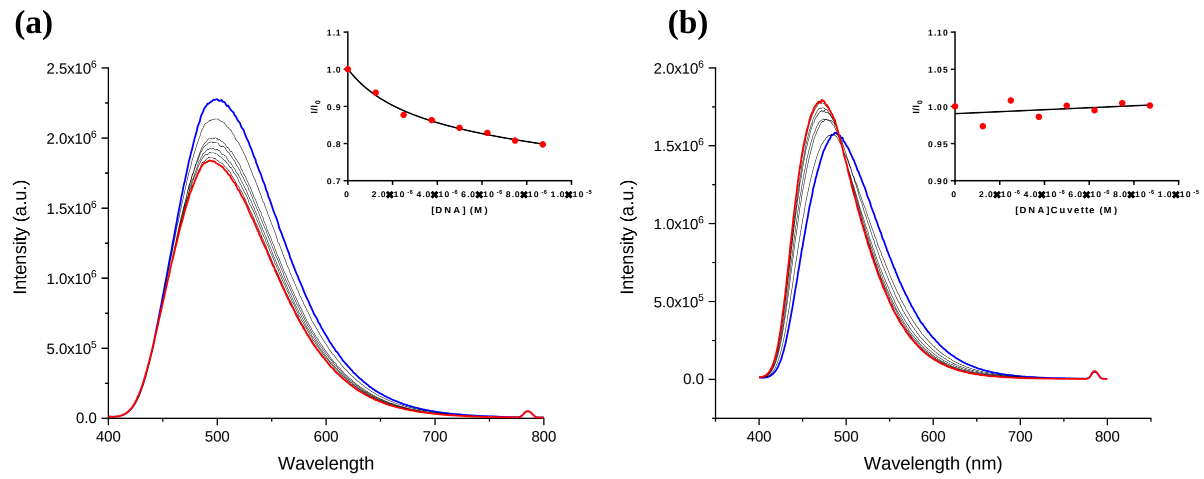

Figure S18. Fluorimetric titrations of (a) Spiro-NMe3 (**1**) and (b) Spiro-SO3 (**2**) with HSA in Tris 10 mM, KCl 100 mM, pH= 7.4, [Ligand] = 3 μM. Insets: Plot of I/I_0 *vs.* [DNA].

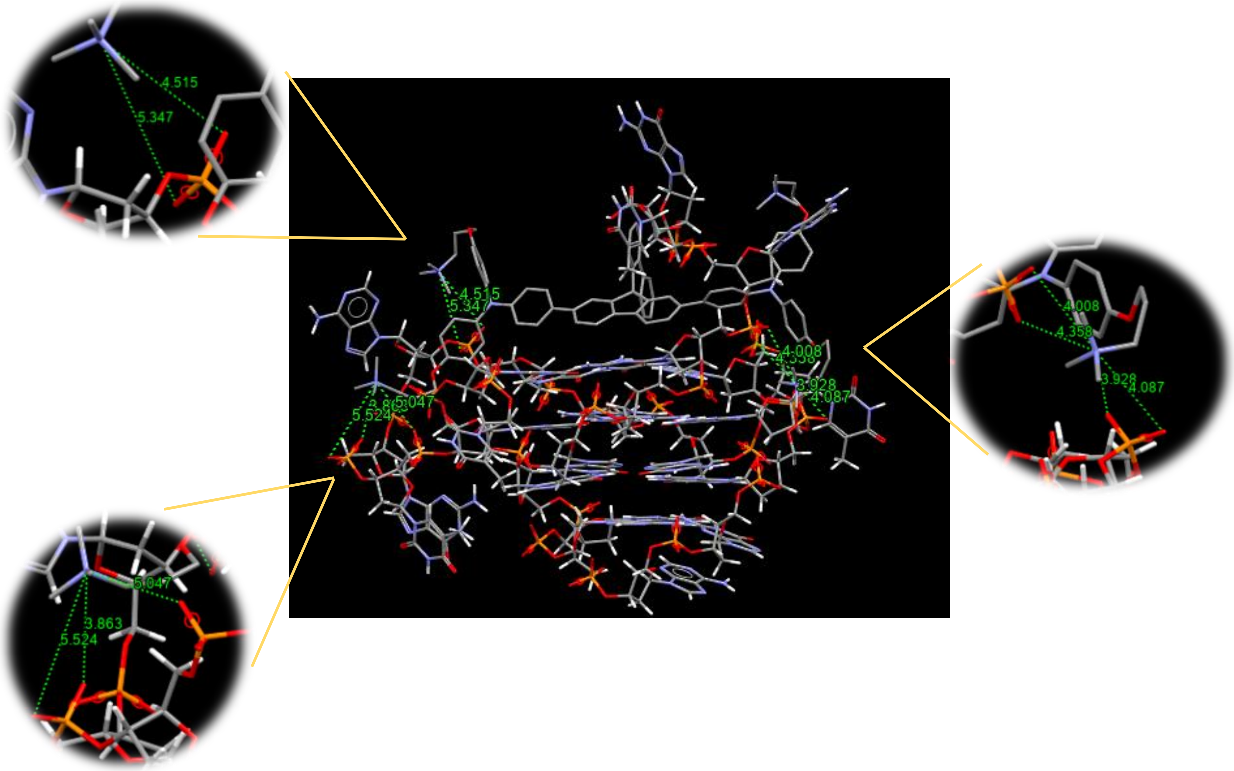

Figure S19. Minimum energy conformer for the interaction of Spiro-NMe3 (**1**) with G-quadruplex (PDB: 2MGN). Insets: Hydrogen and electrostatic interactions formed.

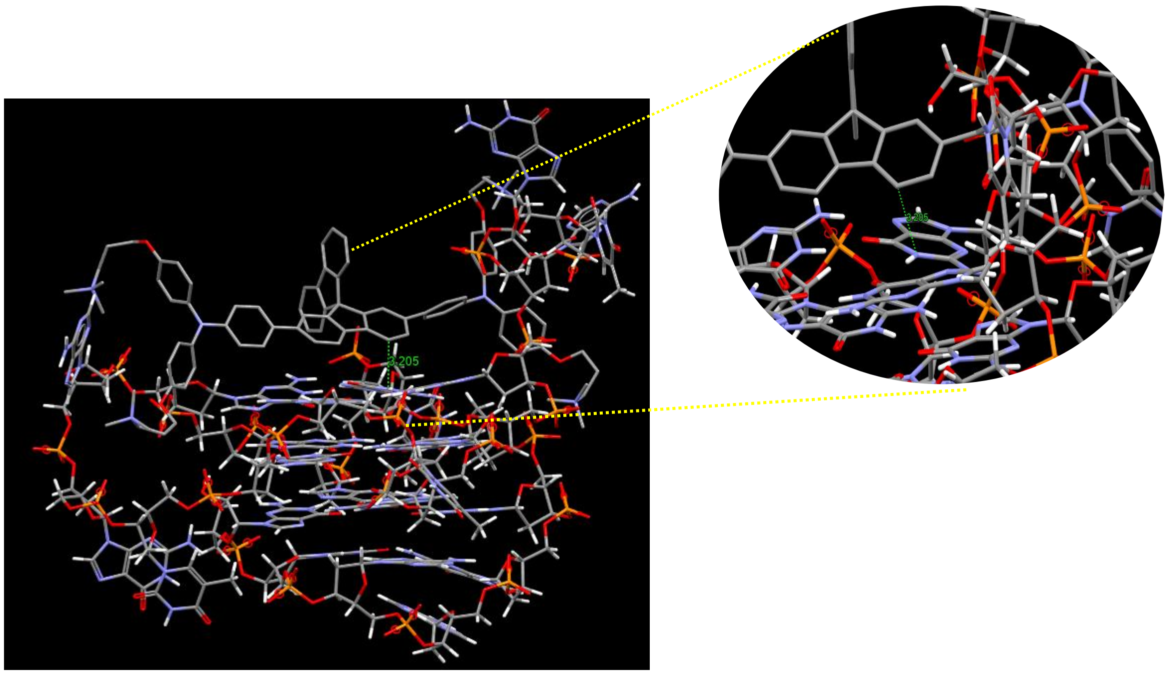

Figure S20. Minimum energy conformer for the interaction of Spiro-NMe3 (**1**) with G-quadruplex (PDB: 2MGN). Insets: Distance calculated between the G-quartet and fluorene moiety.

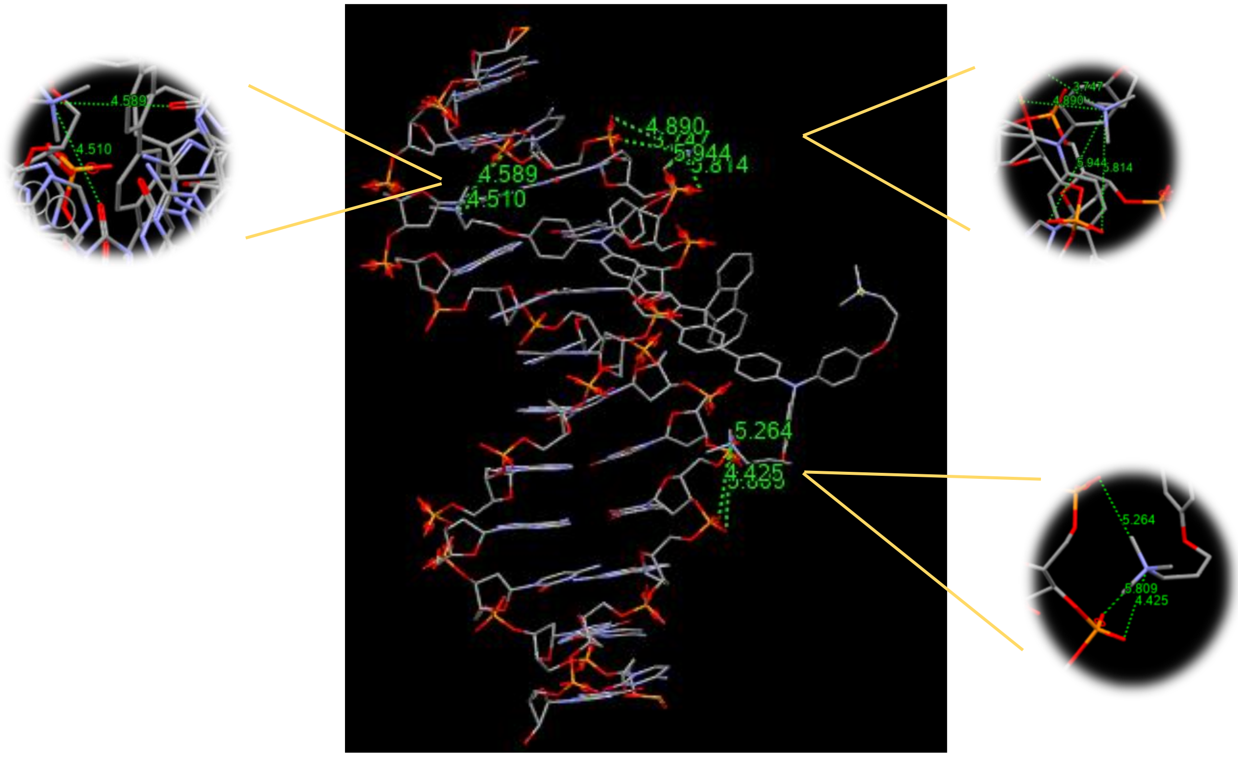

Figure S21. Minimum energy conformer for the interaction of Spiro-NMe3 (**1**) with duplex (PDB: 296D). Insets: Hydrogen and electrostatic interactions formed.

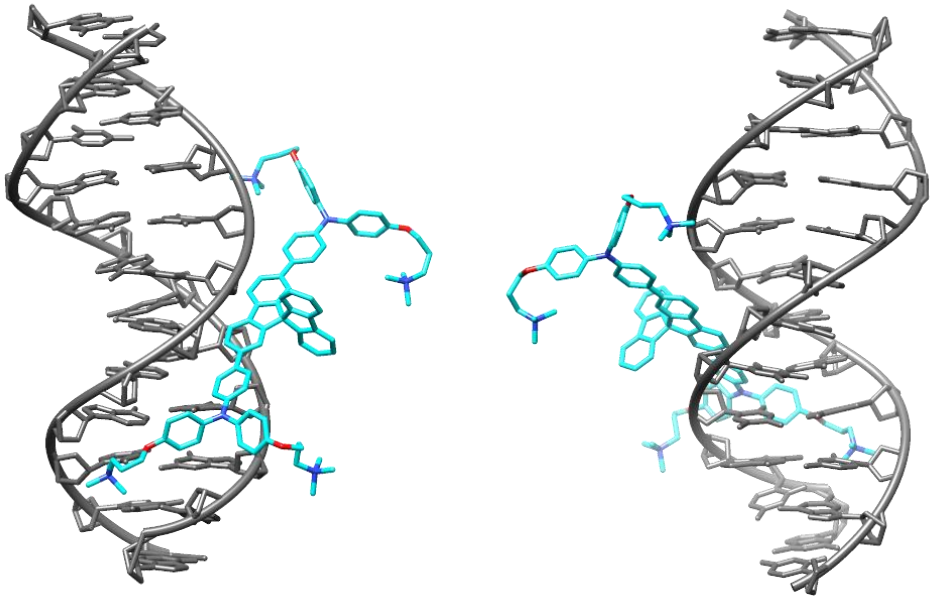

Figure S22. Different views of the minimum energy conformer for the interaction of Spiro-NMe3 (**1**) with duplex (PDB: 296D).

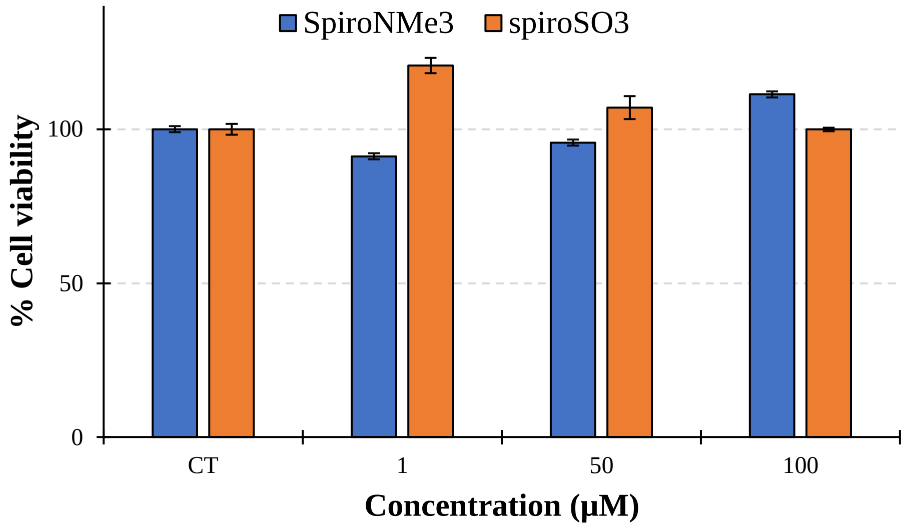

Figure S23. Cell viability assay in HeLa cells of Spiro-NMe3 (**1**) and Spiro-SO3 (**2**) at different concentrations and 24h incubation. (CT: control).

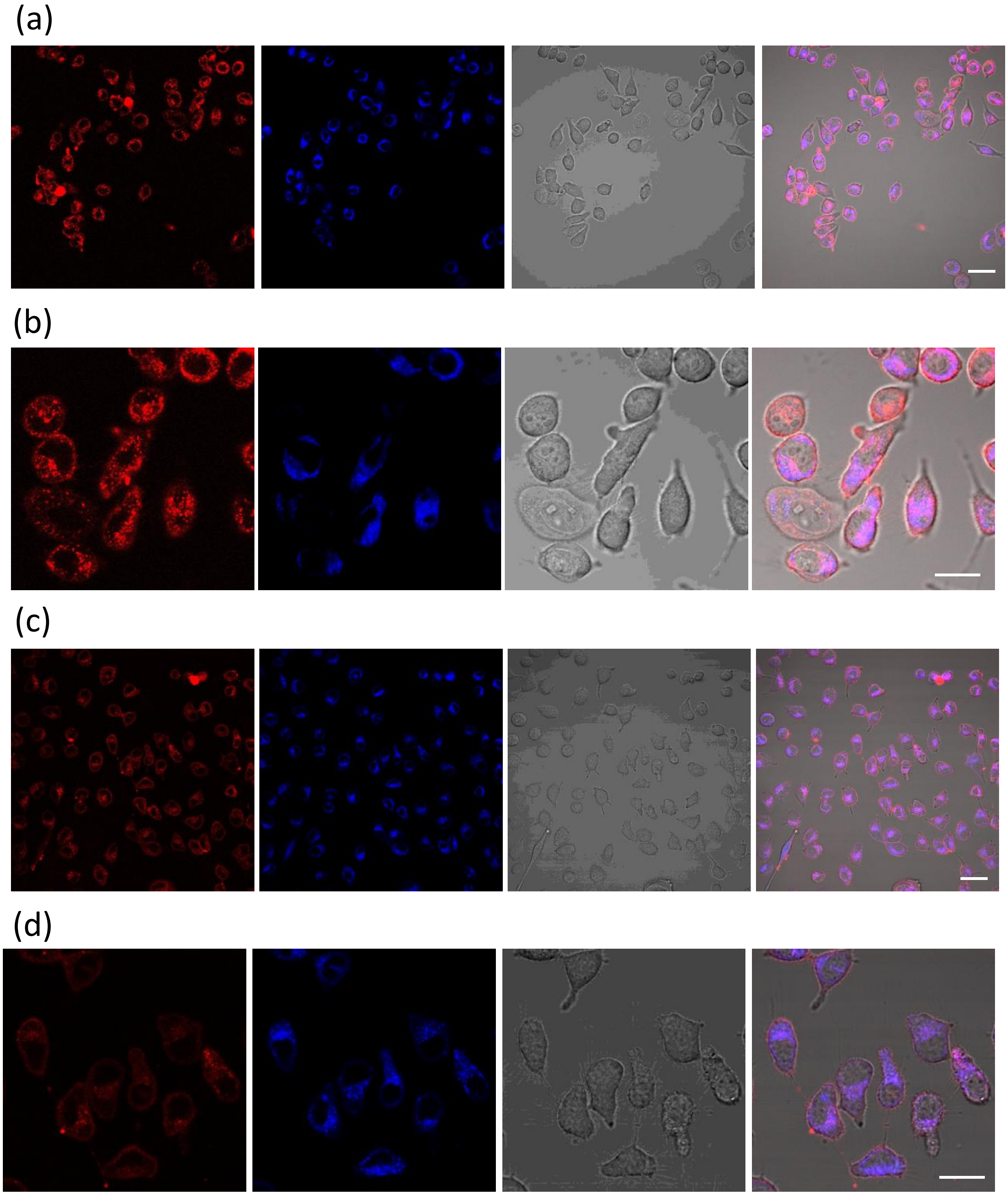

Figure S24. Confocal fluorescence images of HeLa cells incubated with Spiro-SO3 (20 µM, 2h) and Mitotracker DR (a and b) or Lysotracker DR (c and d). Extreme left panel is the fluorescence emission of the ligands (λ_{exc} = 420 nm), central left panel is fluorescence emission of Deep Red dyes (λ_{exc} = 650 nm), central right panel is bright field and extreme right panel is the image emerged from fluorescence and bright field. Bar, 20 µm.

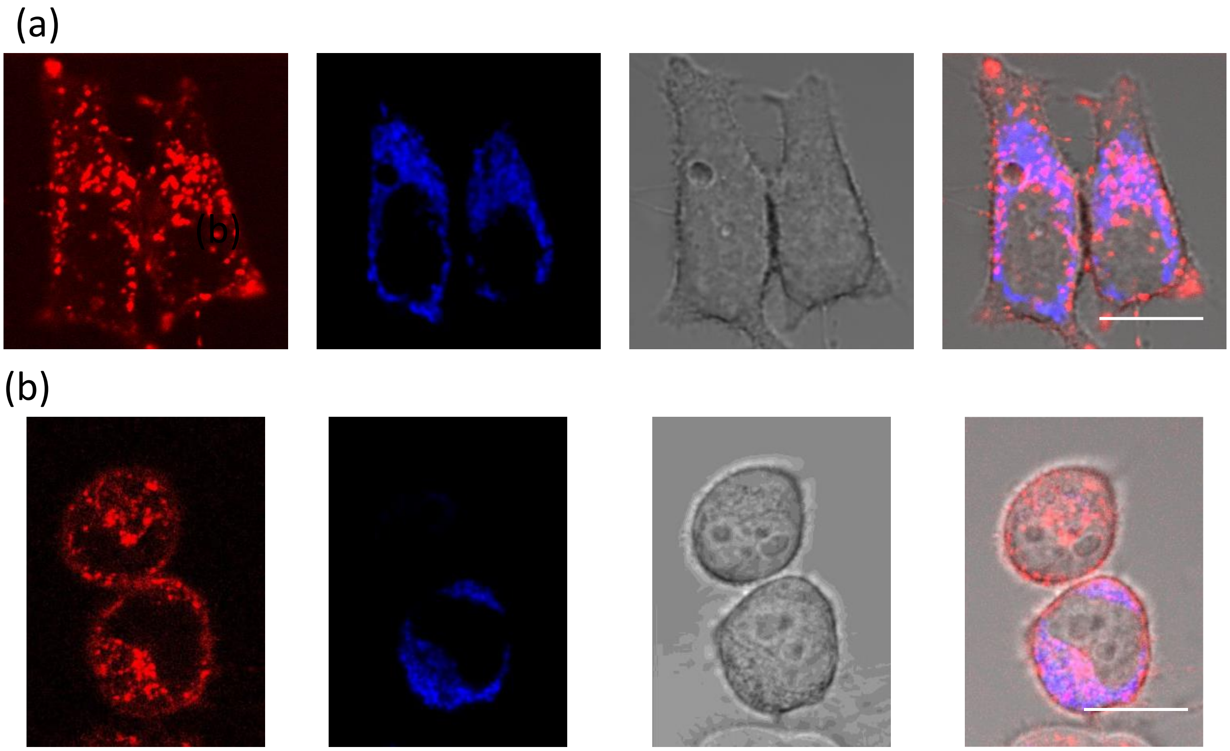

Figure S25. Confocal fluorescence images of HeLa cells incubated with Spiro-NMe3 (20 µM, 2h) and Mitotracker DR (a) or Spiro-SO3 (20 µM, 2h) and Mitotracker DR (b). Extreme left panel is the fluorescence emission of the ligands (λ_{exc} = 420 nm), central left panel is fluorescence emission of Deep Red dyes (λ_{exc} = 650 nm), central right panel is bright field and extreme right panel is the image emerged from fluorescence and bright field. Bar, 20 µm.

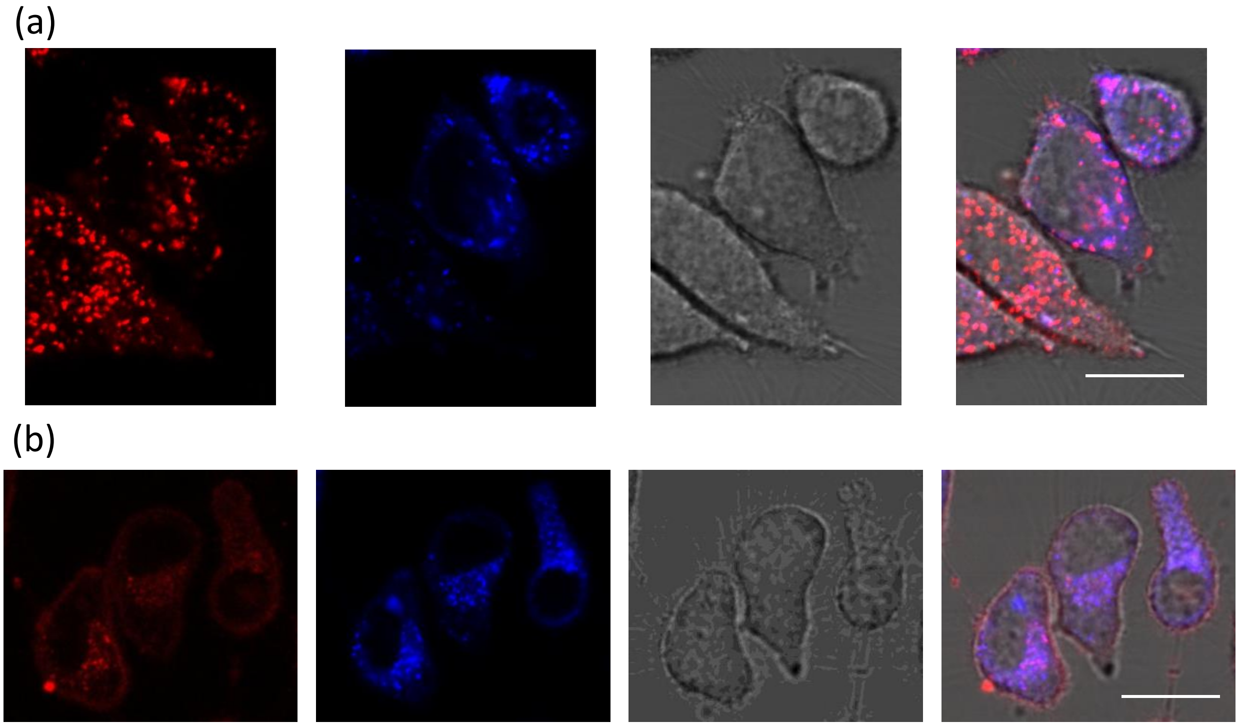

Figure S26. Confocal fluorescence images of HeLa cells incubated with Spiro-NMe3 (20 µM, 2h) and Lysotracker DR (a) or Spiro-SO3 (20 µM, 2h) and Lysotracker DR (b). Extreme left panel is the fluorescence emission of the ligands (λ_{exc} = 420 nm), central left panel is fluorescence emission of Deep Red dyes (λ_{exc} = 650 nm), central right panel is bright field and extreme right panel is the image emerged from fluorescence and bright field. Bar, 20 µm.
